# mRNA-based precision targeting of neoantigens and tumor-associated antigens in malignant brain tumors

**DOI:** 10.1186/s13073-024-01281-z

**Published:** 2024-01-25

**Authors:** Vrunda Trivedi, Changlin Yang, Kelena Klippel, Oleg Yegorov, Christina von Roemeling, Lan Hoang-Minh, Graeme Fenton, Elizabeth Ogando-Rivas, Paul Castillo, Ginger Moore, Kaytora Long-James, Kyle Dyson, Bently Doonan, Catherine Flores, Duane A. Mitchell

**Affiliations:** 1 University of Florida, 1333 Center Drive, BSB B1-118, Gainesville, FL 32610 USA; 2 Boston Medical Center, Boston, MA USA

**Keywords:** Personalized immunotherapy, Cancer immunity, mRNA therapeutics, Vaccines, Adoptive T cell therapy, Immune checkpoint blockade, Brain tumors, Glioblastoma

## Abstract

**Background:**

Despite advancements in the successful use of immunotherapy in treating a variety of solid tumors, applications in treating brain tumors have lagged considerably. This is due, at least in part, to the lack of well-characterized antigens expressed within brain tumors that can mediate tumor rejection; the low mutational burden of these tumors that limits the abundance of targetable neoantigens; and the immunologically “cold” tumor microenvironment that hampers the generation of sustained and productive immunologic responses. The field of mRNA-based therapeutics has experienced a boon following the universal approval of COVID-19 mRNA vaccines. mRNA-based immunotherapeutics have also garnered widespread interest for their potential to revolutionize cancer treatment. In this study, we developed a novel and scalable approach for the production of personalized mRNA-based therapeutics that target multiple tumor rejection antigens in a single therapy for the treatment of refractory brain tumors.

**Methods:**

Tumor-specific neoantigens and aberrantly overexpressed tumor-associated antigens were identified for glioblastoma and medulloblastoma tumors using our cancer immunogenomics pipeline called *O*pen *R*eading Frame *A*ntigen *N*etwork (O.R.A.N). Personalized tumor antigen-specific mRNA vaccine was developed for each individual tumor model using selective gene capture and enrichment strategy. The immunogenicity and efficacy of the personalized mRNA vaccines was evaluated in combination with anti-PD-1 immune checkpoint blockade therapy or adoptive cellular therapy with ex vivo expanded tumor antigen-specific lymphocytes in highly aggressive murine GBM models.

**Results:**

Our results demonstrate the effectiveness of the antigen-specific mRNA vaccines in eliciting robust anti-tumor immune responses in GBM hosts. Our findings substantiate an increase in tumor-infiltrating lymphocytes characterized by enhanced effector function, both intratumorally and systemically, after antigen-specific mRNA-directed immunotherapy, resulting in a favorable shift in the tumor microenvironment from immunologically cold to hot. Capacity to generate personalized mRNA vaccines targeting human GBM antigens was also demonstrated.

**Conclusions:**

We have established a personalized and customizable mRNA-therapeutic approach that effectively targets a plurality of tumor antigens and demonstrated potent anti-tumor response in preclinical brain tumor models. This platform mRNA technology uniquely addresses the challenge of tumor heterogeneity and low antigen burden, two key deficiencies in targeting the classically immunotherapy-resistant CNS malignancies, and possibly other cold tumor types.

**Supplementary Information:**

The online version contains supplementary material available at 10.1186/s13073-024-01281-z.

## Background

Immunotherapy has transformed the prospects of cancer treatment, with immune checkpoint inhibitors (ICIs) and adoptive cellular therapy (ACT) exhibiting impressive success in curing a multitude of cancer types, particularly melanoma and hematological malignancies [1–5]. Despite these advances, solid tumors such as glioblastoma (GBM) and medulloblastoma (MB) remain challenging to treat with immunotherapy [6–11]. mRNA-based immunotherapeutics have emerged as a promising research area, particularly following the accelerated approval and effectiveness of COVID-19 mRNA vaccines [12–15]. Our group has previously demonstrated the potential of mRNA-based therapeutics in combination with ACT and the relevance of mRNA-loaded dendritic cell (DC) vaccines in clinical and preclinical studies of brain tumors [16–20].

Tumor heterogeneity is a hallmark of GBM and MB resulting in immune resistance and escape [10]. Recent human studies have also shown evidence of tumor immunoediting and loss of antigen targets in GBM after monoclonal chimeric antigen receptor (CAR) T-cell therapy [9]. To overcome these limitations, it is imperative to investigate the utilization of the full spectrum of tumor antigens, which includes both neoantigens and tumor-associated antigens (TAAs) [21]. Vaccination strategies can introduce a diverse array of tumor antigens and activate tumor antigen-specific T-cells systemically to enhance immune response at the tumor site [21, 22]. To achieve this, mRNA-based vaccines exhibit unique advantages over other modalities. First, mRNA allows simultaneous delivery of multiple tumor antigens, making them more resistant to immunoediting and antigen loss [14, 23]. Second, mRNA can readily encode for full-length tumor antigens, allowing the antigen-presenting cells (APCs) to simultaneously present and cross-present multiple epitopes [14, 23]. Third, mRNA have the potential to induce a broader T-cell response without being restricted by human leukocyte antigen (HLA) types, which is often a limitation in peptide-based vaccine trials [14, 23, 24]. Finally, the production of synthetic mRNA is rapid, tunable, and cost-effective, supporting the production of on-demand vaccines [23].

Advances in genome-wide sequencing technology have highlighted the unique genetic makeup of individual tumors and the importance of personalized immunotherapy to treat patients. However, the translation of personalized therapeutic vaccines into human trials has been hindered by significant technical requirements. Nevertheless, recent advancements have led to the initiation of clinical evaluations of neoantigen-specific vaccines, marking a new era in therapeutic tumor vaccines [25, 26]. The use of sequence-defined mRNA for personalized vaccines is attractive for treating characteristically “cold” tumors such as GBM and MB, and for developing target-specific therapeutics that go beyond traditional pharmacological agents [14, 27].

One important consideration for personalized cancer vaccines is the identification of immunogenic antigens with epitopes that have strong binding affinity to patients’ HLA molecule. We have established an immunogenomics pipeline, known as the *O*pen *R*eading Frame *A*ntigen *N*etwork (O.R.A.N), for identifying immunogenic antigens, including neoantigens and TAAs. Furthermore, we developed a selective gene enrichment platform for the production of tumor antigen-specific mRNA-therapeutic referred to as TOFU (Tumor Open reading Frames that are Unique) mRNA that encode a large number of tumor antigens, ranging from 20 to 300, in a single vaccine. Our customizable TOFU mRNA-therapeutic platform leverages the in vitro transcription (IVT) mRNA technology to provide virtually unlimited amounts of antigens for each tumor, enabling the development of personalized mRNA vaccines with unprecedented efficiency and affordability while overcoming the need for procurement of large tissue samples from patients, which is often a major hurdle. We have conducted experiments to assess the feasibility of producing TOFU mRNA-therapeutics for murine and human GBM and MB tumors and validated the immunogenic properties of the predicted antigens. We also demonstrate the efficacy of the TOFU mRNA-loaded DC vaccines (TOFU mRNA vaccines) in combination with standard immunotherapy approaches, such as ICI and ACT, in multiple preclinical brain tumor models. Our findings indicate that the use of TOFU mRNA-directed immunotherapy leads to improved survival in GBM hosts by stimulating T-cell activation and effector function and by altering the immunosuppressive tumor microenvironment as determined using sequencing techniques including single-cell RNA sequencing (RNA-seq) and TCR-seq. In conclusion, we have established a personalized and customizable mRNA-therapeutic that effectively targets multiple tumor antigens simultaneously to generate a potent anti-tumor response in traditionally immunotherapy-resistant CNS malignancies.

## Methods

### Mice

Female 4- to 8-week-old C57BL/6 mice (Jackson Laboratories stock 000664) were used for all experiments. The investigators adhered to the “Guide for the Care and Use of Laboratory Animals” as proposed by the Institute for Laboratory Animal Research, National Research Council. All study experiments were reviewed and approved by the University of Florida Institutional Animal Care and Use Committee (IACUC) under protocol ID 202100000029. The husbandry practices and animal care such as providing analgesics, moist food, or saline, were conducted according to the guidelines mentioned under this protocol. The animals were euthanized using CO2 as per the institutional guidelines in all experiments for tissue collection or upon reaching a humane endpoint.

### Tumor models

KR158B-luc (Kluc) glioma line (provided by Dr. Karlyne M. Reilly, NCI Rare Tumor Initiative, NIH) and GL261 (DCTD Tumor Repository, NCI) cells have been verified histologically as high-grade glioma, and gene expression analysis confirmed appropriate haplotype background and expression of astrocytoma-associated genes [28]. The tumor cells were cultured in DMEM high-glucose media (Fisher-Scientific), 10% FBS (VWR), 1% Penn-strep (Fisher Scientific), and tested annually for pathogens by IDEXX BioResearch (Westbroon, ME). The cells were passaged in vitro less than 4 times after thawing. Ptch MB line was provided in collaboration with Dr. Robert Wechsler-Reya at the Sanford Burnham Research Institute, La Jolla, CA. This line is maintained in vivo and checked annually for genetic markers consistent with sonic hedgehog molecular subtype medulloblastoma [29]. Neural stem cell (NSC) tumor cells were generated through previously described in vitro culture of sorted granule neuron precursor cells [29].

### Tumor implantation

For tumor inoculation, 2 × 10^4^ Kluc and GL261 cells were suspended in 50% methylcellulose (Fisher-Scientific) and 50% PBS (Fisher-Scientific) and implanted into the caudate nucleus 3.5 mm deep and 2 mm lateral to the bregma using a stereotactic frame (Stoelting, cat no. 53311) and a 25-gauge needle [17]. NSC cells (1 × 10^3^) and Ptch cells (1.25 × 10^5^) were implanted into the cerebellum 1 mm lateral to the midline and 3 mm deep [29].

### Tumor isolation and sequencing

Untreated Kluc, GL261, NSC, or Ptch tumors (*n* = 3) were harvested 3 weeks post implantation. Quick-DNA/RNA Miniprep Plus Kit (Zymo Research Corp) was used to isolate DNA and RNA from all samples. RNA and whole exome sequencing (WES) were performed (Novogene) and the gene expression data was applied to the O.R.A.N pipeline for the identification of tumor antigens.

### O.R.A.N pipeline for neoantigen and TAA prediction

O.R.A.N is an immunogenomics pipeline capable of predicting neoantigens including single nucleotide variations (SNVs) and insertions/deletions (indels), as well as TAAs, including cancer-testis and developmental antigens expressed in tumor cells. The workflow of the pipeline includes the identification of mutations and tumor-associated genes, annotation and HLA-binding prediction, and a peptide-similarity filter to remove any non-unique antigens. For murine antigen prediction, tumor RNA-seq reads were aligned to the Ensemble 99 Gencode M24 reference genome (GRCm38), and transcript expression was quantified with the RSEM algorithm [30]. Murine SNVs and indels were called from the WES data and confirmed with the RNA-seq data. Tumor mutations were called by mutect2-GATK4 pipeline [31]. Only RNA-expressed SNVs and indels were used as input for peptide-MHC (pMHC) affinity prediction. MHC-I and MHC-II haplotypes of C57BL/6 mice were acquired from the Jackson Laboratory. Tumor-associated antigens (TAA) were defined from the RNA-seq data using a transcript per million (TPM) threshold of > 1 in tumor and < 1 across normal tissues (*n* = 95, ENCODE v3). To ensure the identification of TAAs is stringent and truly unique to the tumors, we considered any gene expression as the sum of expression of all transcripts (or isoforms) of that gene and applied the threshold of gene TPM expression < 1 in the normal tissues and transcript TPM expression > 1 in tumors. Peptide binding affinity was estimated using the pVAC-Seq pipeline as implemented in pVACtools version 1.55 [32, 33]. MHC-I restricted epitopes were filtered in a step-wise fashion using the following parameters: predicted mutant peptide sequence binding affinity < 500 nM, variant allele fraction (VAF) > 0.6, gene expression value of > 1 TPM. MHC-II restricted peptides were filtered using the same parameters except that a binding affinity cutoff score of 1000 nM was used. To ensure tumor-specificity, a peptide similarity filter was applied to all MHC-selected peptide epitopes which were screened against a customized murine proteomic library and proteome database (Ensembl v99) to guarantee that epitopes were not shared by other expressed isoforms or genes. For TOFU antigen-specific mRNA library prep, all unique antigens expressed by each tumor sample (*n* = 3 per tumor model) were included. For human antigen prediction, five primary GBM patients were chosen at random from the TCGA cohort: TCGA-06–0125-01A, TCGA-06–0190-01A, TCGA-06–0211-01B, TCGA-14–1034-01A, and TCGA-19–4065-01A. Genomic alignments (using GRCh38 d1) were downloaded and then divided into fastq data. Mutation Annotation Format (MAF) files for these patients were obtained from the MC3 0.2.8 controlled dataset, converted, and annotated into VCF files using the maf2vcf tool and vep 93 (GRCh37) [34]. Subsequently, mutations were filtered based on read count, using the new RNAseq alignment (GRCh37, hs37d5). Tumor overexpressed genes were identified by their transcription expression being > 1 TPM in the tumor tissue and < 1 TPM in normal tissues, as determined by data from the GTEx dataset. The haplotypes for HLA-I and II of the patients were identified from the aforementioned fastq files using Optitype and Phlat [35, 36]. For the prediction of epitopes, 8–11 mer MHC-I epitopes and 15 mer MHC-II epitopes were computed using pvactools 4 [33]. The subsequent peptide filtering was similar to the murine pipeline. The complete list of antigens for the GBM patients’ tumors can be found in Additional file 2.

### Tumor antigen-specific TOFU mRNA library generation

cDNA libraries were constructed from total tumor RNA (ttRNA) isolated from the tumors using SMARTScribe™ Reverse Transcriptase (Takara Bio), Advantage 2 Polymerase Mix (Takara Bio), oligo dT primer, and TSO-T7 primer with T7 promoter sequence to support in vitro mRNA transcription (IVT). The identified TOFU antigens were subjected to a gene enrichment strategy which included hybridization and capture with antigen-specific probes (probes were designed to target specific transcripts) (SureSelect custom library probes and SureSelectXT reagent kit, Agilent Technologies), and PCR amplification from the total tumor cDNA libraries. TOFU mRNA was prepared from the post-enrichment cDNA pool using mMESSAGE mMACHINE T7 in vitro transcription kit (Fisher Scientific) and Poly(A) Tailing kit (ThermoFisher). The TOFU mRNA libraries were validated for the enrichment of antigen-specific genes using qPCR (PrimeTime Gene Expression Master Mix and primers, IDT DNA Inc.) and RNA-seq (UFHCC, Next Generation Sequencing Core) for all 4 tumor models before performing subsequent experiments. The volcano plot figures were generated using the Ggplot2 program (H. Wickham. Ggplot2: Elegant Graphics for Data Analysis. Springer-Verlag New York, 2016).

### Human tumor-specific gene selection

We performed RNA-seq analysis of a primary human GBM tumor sample (FCBTR) and identified tumor-specific and associated genes. We selected genes that were uniquely upregulated in the GBM tumor but were absent in the normal brain as well as genes containing non-synonymous SNVs as detected using DNASTAR. A total of 316 genes containing non-synonymous SNVs were identified for which a gene-specific library construction was done similarly to the murine models using customized probes and gene amplification.

### Dendritic cell generation and vaccination

Briefly, bone marrow was harvested from 4 to 5-weeks-old C57BL/6 mice and red blood cells were lysed using RBC lysis buffer (BioLegend), following which mononuclear cells including the myeloid progenitors were cultured in RPMI medium supplemented with GM-CSF (10 ng/ml, R&D) and IL-4 (10 ng/ml, R&D) for 9 days. On day 8 of the culture, mature DCs were electroporated (BTX Harvard Apparatus, ECM 830) using electroporation cuvettes (BTX Harvard Apparatus, 2-mm gap) with 10 μg of TOFU RNA and cultured overnight. For priming, 2.5–5 × 10^5^ TOFU mRNA-pulsed DCs were injected intra-dermally into naïve animals. Control DCs (Ctl DCs) for all experiments were DCs electroporated with no RNA but cultured in the same conditions as the TOFU mRNA-pulsed DCs.

### TOFU antigen-reactive lymphocyte expansion and antigen re-challenge assay

We screened for 12 Kluc, 20 GL261, 20 NSC, and 20 Ptch-specific TOFU antigen peptides (Genemed Synthesis Inc, custom peptides) that were predicted to be immunogenic in nature and had a high binding affinity to HLA-Kb or HLA-Kd (complete list of peptides used can be found in Additional file 3). Several of these antigens were shared between multiple tumor models (shared TAAs between NSC and Ptch tumors) and showed immunogenicity in each model. Briefly, one-week post priming with TOFU mRNA-pulsed DCs, splenocytes were harvested and co-cultured with individual predicted immunogenic peptides. For expansion, 4 × 10^6^ splenocytes were plated in 24 well plates with a 10-μg/ml concentration of the target peptide. On the following day, IL-2 (25 U/ml, R&D) was supplied to the splenocytes and allowed to expand for 4 days. On day 5, the cells are washed and resuspended in media without peptide or IL-2 for a day. Re-challenge assay was conducted with the target antigen peptide, a reference peptide or DMSO at a concentration of 1 μg/ml in 96-well U-bottom plates (*n* = 3 per condition). IFN-γ secretion was determined using ELISA Max Deluxe Set Mouse IFNγ (Biolegend) from the harvested and frozen supernatants following 24 h of culture. Antigen-specific responses were determined as IFN-γ secretion over 100 pg/ml concentration and at least a 2-fold increase over the response to the irrelevant peptide**.**

### Treatment with TOFU mRNA vaccines and immune checkpoint inhibition

Kluc or GL261 GBM tumor-bearing mice were treated starting on day 6 after the intracranial injection. The cohort of mice used in this experiment were mice that received no treatment (Untreated), mice which received control unloaded DCs in combination with anti-PD-1 Ab (Ctl-DCs + PD-1) or IgG Ab (Ctl-DCs + IgG), and mice which received TOFU mRNA-pulsed DCs in combination with anti-PD-1 Ab (TOFU-DCs + PD-1) or IgG Ab (TOFU-DCs + IgG) (*n* = 7 mice per group). Briefly, 5 × 10^5^ TOFU-DCs or Ctl DCs were injected intradermally along with anti-PD-1 or IgG Abs (10 mg/kg) (BioXcell, clones RMP1-14 and 2A3, respectively) delivered intraperitoneally. The DC vaccines were administered weekly for 3 weeks for a total of 3 vaccines. The anti-PD-1 and IgG Abs were administered to the mice every 3rd day for 2 weeks for a total of 5 doses. Bioluminescent imaging of the Kluc tumors was performed using luciferin substrate (Perkin Elmer LAS Inc) and the IVIS Spectrum Imaging System at the end of the treatment regimen on day 20 post the tumor implantation (*n* = 5 mice per group). For RNA-seq analysis of the tumor microenvironment, the animals were euthanized on day 21 after the tumor implantation (*n* = 4–5 mice per group) and the tumor tissue was processed for downstream analysis (see the “Tumor dissociation” section of the methods).

### TOFU antigen-reactive lymphocyte expansion for adoptive cellular therapy

Splenocytes from primed animals were harvested and co-cultured with TOFU RNA-pulsed DCs and IL-2 (50 U/ml, R&D) for 5 days. For in vitro re-challenge assays, TOFU antigen-specific T-cells were co-cultured with APCs electroporated with TOFU mRNA or GFP mRNA or Kluc tumor cells in a 10:1 ratio in 96-well U-bottom plates in triplicates. IFN-γ secretion was determined from the harvested and frozen supernatants following 48 h of culture. For ACT, Kluc or GL261 GBM tumor-bearing mice were divided into three treatment groups- mice that received no treatment (Untreated), mice that received 9-Gy radiation and HSC rescue (9 Gy-TBI), and mice that received 9-Gy radiation, HSC rescue, and ACT transfer along with TOFU mRNA vaccine therapy (TOFU-ACT) (*n* = 7–9 mice per group). The treatment of tumor-bearing mice began with 9-Gy x-ray myeloablation on day 5 after intracranial injection, following which mice received 5 × 10^4^ lineage-depleted HSCs (Miltenyi Biotec) via a single intravenous injection in the tail vein on day 6. In addition, the TOFU-ACT cohort also received 5–8 × 10^6^ ex vivo expanded TOFU antigen-specific T-cells on day 6 with the HSCs and 2.5 × 10^5^ TOFU mRNA-pulsed DC vaccines intradermally weekly for 3 weeks beginning day 7. Bioluminescent imaging of the Kluc tumors was performed as described above at the end of the treatment regimen on days 21 and 32 post the tumor implantation (*n* = 5 mice per group). For single-cell RNA-seq and TCR-seq analysis of the tumor microenvironment, the animals were euthanized on day 21 after the tumor implantation (*n* = 4 mice per group) and tissue was processed according to the ‘tumor dissociation’ part of the methods.

### Tumor dissociation

For all experiments involving tumor-infiltrating immune cell phenotyping and sequencing, tumors were isolated 2 days after the final DC vaccine. Briefly, the right cerebral cortex (where the tumor is implanted) was excised and processed using the Miltenyi Multi Tissue Dissociation Kit and Debris Removal solution (Miltenyi Biotech). CD45 + cell selection was performed using CD45 (TIL) MicroBeads for mice (Miltenyi Biotech) and cells were counted and suspended in FACs buffer before proceeding with the next assay. The viability of cells after the isolation process was typically over 90%.

### Flow cytometry

Flow cytometry was performed on the BD Biosciences FACS Symphony and Canto. The antibodies used were- anti-CD45 APC-Cy7 (clone 30-F11), anti-CD3 FITC (clone 17A2), anti-CD4 PE (clone GK1.5), anti-CD8 BV570 (clone 53–6.7), anti-CD62L APC (clone MEL-14), anti-CD44 BV786 (clone IM7), anti-PD-1 BV421 (clone 29F.1A12), and live/dead PI dye (all from Biolegend). Antibodies were applied as per the manufacturer's recommendation and processed in FACs buffer consisting of 2% FBS in PBS. Analysis and flow plots were generated with FlowJo version 10 (Tree Star) after the omission of doublets, dead cells, and debris and were gated on size and granularity.

### Immune monitoring with bulk RNA-seq and TCR-seq

CD45 + immune cell selection from the tumor microenvironment was done as described before. Celero™ EZ DNA-Seq, 24 reactions kit (Tecan Genomics) was utilized to generate sequencing libraries following the manufacturer’s instructions. RNA-sequencing was performed using Illumina NovaSeq 6000, SP 2 × 50. For TCR-seq, the sequencing libraries were generated using SMARTer Mouse TCR a/b Profiling Kit (Takara Bio USA) following the manufacturer’s instructions. Only primers specific to the TCR-beta chain were used during the library prep. TCR-sequencing was performed using Illumina MiSeq, 2 × 300. All sequencing tasks were completed at the UFHCC Next Generation Sequencing Core. TCR vb data alignment and analysis were performed using MixCR 4.0 [37]. TCR data visualization and graphs were prepared using GraphPad Prism 8.0. Bulk RNA-seq fastq data was aligned and quantified using RSEM [30]. Immune cell deconvolution and the pathways enrichment analysis were performed using the Immgen dataset and the NanoString nCounter Immunology pathway through the GSVA tool [38]. Raw data can be found at NCBI GEO [39–41].

### Single-cell RNA sequencing and quality control

For the single-cell RNA-seq experiment, we looked at CD45 + immune cells and the CD45 − flow-through cells after the bead selection following the CD45 + cell isolation process as described above. Mice treated with TOFU-ACT, 9 Gy-TBI, or no treatment (*n* = 4) were used for this experiment. The Chromium Next GEM Single Cell 3ʹ Reagent Kits v3.1 (Dual Index, 10 × Genomics) was utilized to construct the cDNA sequencing libraries. The samples were multiplexed and prepared as per the manufacturer’s instructions and sequenced by Illumina Novaseq 6000. BCL files were demultiplexed to fastq data using Cellranger 7.0 makefastq and were aligned to a customized Luciferase-mm10 mouse genome and counted using Cellranger 7.0 multi. Cells were further demultiplexed using Seurat 4.0 HTOdemux [42]. Samples with over 70% probability of certain Cell Multiplexing Oligo assignment and less than 50% multiplet rate were used for downstream analysis. Genes expressed across at least 3 cells were retained. Cells with over than 250 genes, 500 UMIs, 0.8 Complexity (log10 gene counts/log10 UMI count), and less than 5% mitochondrial genes were selected using Seurat 4.0 [43]. Raw data can be found at NCBI GEO [44].

### Single-cell RNA-seq data analysis

SingleR with built-in Immgen dataset was applied for cell type identification [45]. Differentially expressed genes (DEGs) between cell clusters were extracted using Seurat 4.0 and used for further cell type identification. For CD45 − cells and CD45 + cells, the treatment groups were downsampled to 1000 cells and 15,000 cells each respectively. Cell populations were then visualized using the UMAP algorithm through Seurat 4.0. To visualize gene exhaustion and activations markers, we used the imputation by Alra program [46]. The pheatmap package was used for unsupervised hierarchical clustering to create heatmaps (Kolde, R., Pheatmap: pretty heatmaps. R package version, 1(2), 726.). Pathway enrichment analysis for tumor cells and immune cells was performed with AUCell algorithm using the NanoString nCounter Pan-cancer and Immunology pathway datasets respectively [47]. Interactions between immune populations were analyzed and visualized using the CellChat algorithm [48].

### Statistical Analysis

Statistical tests were performed using GraphPad Prism 8. For T-cell re-challenge and IFN-γ release experiments, 2-way ANOVA with Bonferroni’s multiple comparison test was performed. The flow cytometry data statistics was performed using one-way ANOVA and Dunnett’s multiple comparison tests. For survival experiments, we utilized Mantel–Cox log-rank test. For pathway enrichment and gene expression data analysis, the Kruskal–Wallis test with Dunn’s multiple comparisons was performed. Significance was determined at *p* < 0.05. *P* < 0.05 is *, *p* < 0.01 is **, *p* < 0.001 is ***, and *p* < 0.0001 is ****.

## Results

### Identification of tumor antigens in multiple models of CNS malignancy

**Fig. 1:**
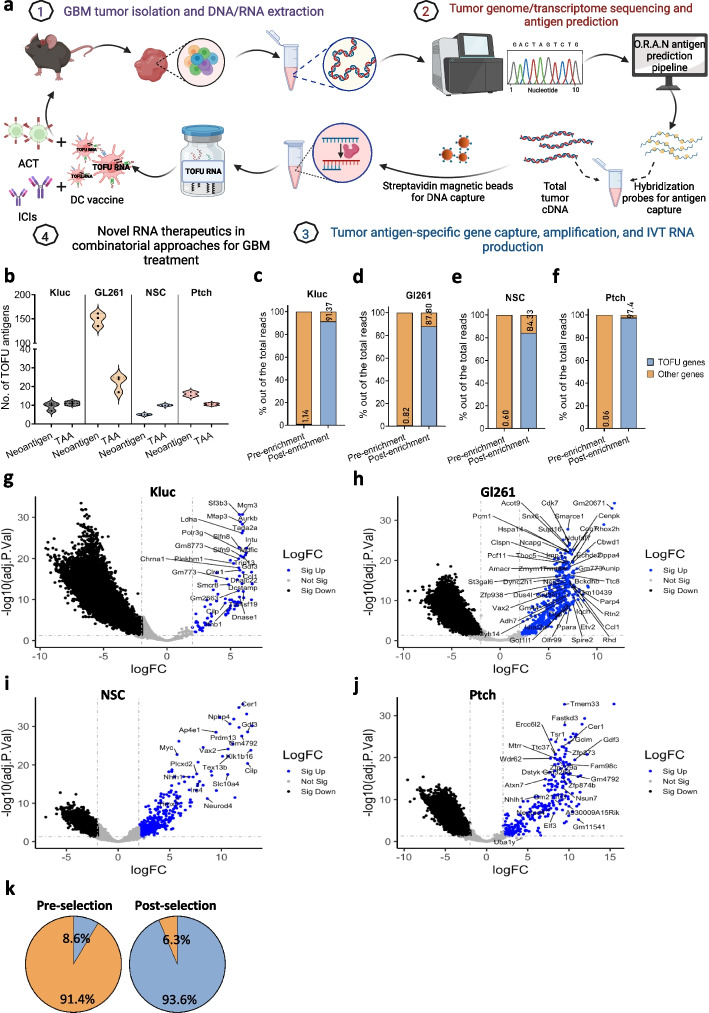
Antigen prediction and tumor antigen-specific TOFU mRNA production. **a** Schematic for tumor antigen-specific TOFU mRNA vaccine development (Created with BioRender.com). **b** The number of neoantigens or tumor-associated antigens (TAAs) predicted for the KR158-Luc (Kluc), GL261, NSC, and Ptch tumors using the *O*pen *R*eading Frame *A*ntigen *N*etwork (O.R.A.N) pipeline (*n* = 3 per tumor). **c**–**f** Enrichment of tumor antigens in TOFU mRNA libraries as compared to total tumor RNA (ttRNA) for the Kluc (**c**), GL261 (**d**), NSC (**e**), and Ptch (**f**) tumors. Blue bars represent the % of TOFU genes and orange represents all other genes in the pool. **g**–**j** Volcano plots demonstrate the fold change increase in the expression of the TOFU antigens following enrichment in Kluc (**g**), GL261 (**h**), NSC (**i**), and Ptch (**j**) tumors. TOFU antigens are highlighted and labeled in each plot for all 4 models respectively. **k** Pie-chart showing enrichment efficiency of tumor-specific and associated genes (316 genes that were selected using differential gene expression and non-synonymous SNVs) from a human GBM patient’s tumor RNA sample. TOFU gene representation is shown in blue and other genes are in orange

Recent progress in cancer immunogenomics has facilitated the identification of tumor-specific antigens by applying comprehensive cancer genomics to antigen discovery [49–51]. We applied this methodology to develop O.R.A.N, an immunogenomics pipeline capable of predicting neoantigens including SNVs and insertions/deletions, as well as TAAs, including cancer-testis and developmental antigens, which are aberrantly over-expressed in tumors cells (Fig. 1a). The identified tumor antigens are referred to as TOFU antigens. A simplified workflow of the pipeline is shown here (Additional file 1: Fig. S1a). The gene expression profile of various glioma models including murine GBM tumors KR158-Luc (Kluc) and GL261 and MB tumors NSC and Ptch was determined using RNA-seq and whole exome sequencing (WES) and was applied to the O.R.A.N pipeline for antigen prediction. Kluc tumors were found to express 45 overexpressed tumor-associated genes and 643 mutations on average, of which 114 were non-synonymous and protein coding (Additional file 1: Fig. S1b–c). After applying the HLA I and II binding affinity filter and a peptide similarity filter to ensure tumor-specificity, 12 unique neoantigens and 15 TAAs were identified (Fig. 1b, Additional file 1: Fig. S1b–c, and Table S1). Similarly, GL261 tumors were found to express 90 overexpressed tumor-associated genes and 6126 mutations on average, of which 1288 were non-synonymous and protein coding (Additional file 1: Fig. S1b–c). After applying the filters,192 neoantigens and 37 TAAs were predicted to be immunogenic targets for GL261 tumors (Fig. 1b, Additional file 1: Fig. S1b–c, and Table S1). NSC tumors were found to express 82 overexpressed tumor-associated genes and 105 mutations on average, of which 32 were non-synonymous and protein coding (Additional file 1: Fig. S1b–c). After applying the filters, 6 neoantigens and 14 TAAs were predicted to be immunogenic targets for NSC tumors (Fig. 1b, Additional file 1: Fig. S1b–c, and Table S2). Similarly, Ptch tumors were found to express 114 overexpressed tumor-associated genes and 1387 mutations on average, of which 224 were non-synonymous and protein coding (Additional file 1: Fig. S1b–c). After applying the filters, 19 neoantigens and 13 TAAs were predicted to be immunogenic targets for Ptch tumors (Fig. 1b, Additional file 1: Fig. S1b–c, and Table S2). Thus, a total of 27 and 229 TOFU antigens were identified for Kluc and GL261 GBM tumors, respectively, while 20 and 32 TOFU antigens were identified for NSC and Ptch MB tumors, as potential targets for vaccine development and antigen-specific T-cell selection.

To determine if the pipeline can predict immunogenic antigens for human tumors, we additionally applied this pipeline to human GBM patients’ tumor samples obtained from TCGA. An average of 7 neoantigens and 132 TAAs were expressed in human GBM tumors which were predicted to be immunogenic and could be leveraged for vaccine therapy (Additional file 1: Fig. S1d).

### The development of TOFU mRNA-therapeutics and enrichment efficiency

We next developed a method for the selective enrichment of specific mRNA species from the total tumor mRNA (ttRNA) pool. This technique allowed us to isolate and enrich selected TOFU antigen mRNA, resulting in the creation of a personalized tumor antigen-specific library based on the genetic alterations of the respective tumors (Fig. 1a). As validated using RNA-seq, the selection resulted in a > 80-fold enrichment of Kluc TOFU antigens from 1.14% in the original ttRNA pool to 91.4% in the enriched TOFU mRNA pool, and > 107-fold enrichment of GL261 TOFU antigens from 0.82 to 87.8% (Fig. 1c–d). Similar results were observed in the MB models, with an > 140-fold enrichment of NSC TOFU antigens from 0.6 to 84.3%, and > 1623-fold enrichment in Ptch TOFU antigens from 0.06 to 97.4% (Fig. 1e–f). RNA-seq analysis of the individual TOFU antigens revealed hundred to several thousand-fold enrichment compared to the baseline expression in all four brain tumor models (Fig. 1g–j). This finding was corroborated using qPCR for a selected number of TOFU antigens in all four models (Additional file 1: Fig. S2a–d). Furthermore, the efficacy of our antigen enrichment methodology was also validated in a patient-derived human GBM tumor sample (from 8.6 to 93.6% antigen expression) (Fig. 1k).

We sought to confirm the capture of full-length gene products, which is imperative for mRNA stability and proper translation into protein and subsequent peptide epitopes on APCs. Using PCR analysis with primers designed to target full-length gene products, we show successful capture for two Kluc neoantigens, *Cilp* (4004 bps*)* and *Smcr8* (2808 bps) (Additional file 1: Fig. S2e), and for a shared TAA expressed by NSC and Ptch tumors, *Neurod4* (size-971 bps) (Additional file 1: Fig. S2f). Additionally, there was no bias towards the size or type of antigen captured, as demonstrated by similar selection efficiencies for antigens of varying lengths (from 419 to 4154 bps) and between neoantigens or TAAs (Additional file 1: Fig. S2g–h).

### Demonstration of antigen-specific responses against TOFU antigens

**Fig. 2:**
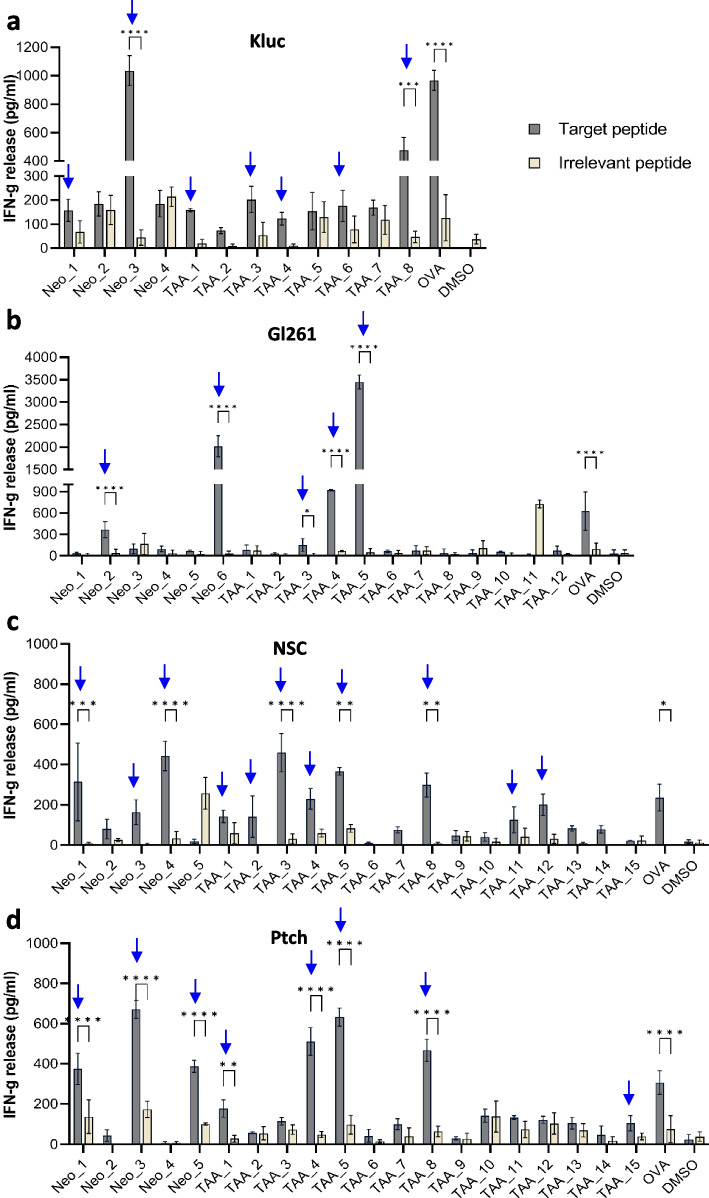
Demonstration of antigen-specific responses against TOFU antigens. **a**–**d** Peptide stimulation and re-challenge of T-cells from Kluc TOFU mRNA primed animals (**a**), GL261 TOFU mRNA primed animals (**b**), NSC TOFU mRNA primed animals (**c**), and Ptch TOFU mRNA primed animals (**d**) with selected predicted neoantigens and TAA peptides (*n* = 3 per sample). IFN-γ release is detected using ELISA after 24 h. Antigen reactivity is considered as > 100 pg/ml IFN-γ production and at least a 2-fold increase over the response to the irrelevant peptide. Statistical analysis is done using 2-way ANOVA with multiple comparisons using Bonferroni correction (two-tailed); *p* < 0.05 is *, *p* < 0.01 is **, *p* < 0.001 is ***, and *p* < 0.0001 is ****. The experiments have been repeated at least 2 times to validate the immunogenicity

To validate the immunogenicity of our predicted tumor antigens, we used dendritic cells (DCs) electroporated with TOFU mRNA as highly effective APCs, capable of eliciting both CD4 + and CD8 + T-cell responses [52, 53]. T-cells primed in vivo by TOFU mRNA-pulsed DCs (TOFU-DCs) and stimulated ex vivo with a subset of peptide-encoded TOFU-specific epitopes were evaluated by IFN-γ secretion, demonstrating reactivity towards Kluc, GL261, NSC and Ptch TOFU antigens. We observed increased IFN-γ production compared to irrelevant peptides for 7 Kluc antigen peptides (Fig. 2a), 5 GL261 antigen peptides (Fig. 2b), 11 NSC antigen peptides (Fig. 2c), and 8 Ptch antigen peptides (Fig. 2d), respectively. These results also confirmed that TOFU mRNA is biologically active and can promote antigen-specific T-cell responses.

### Therapeutic efficacy of TOFU mRNA vaccines in combination with ICI

**Fig. 3:**
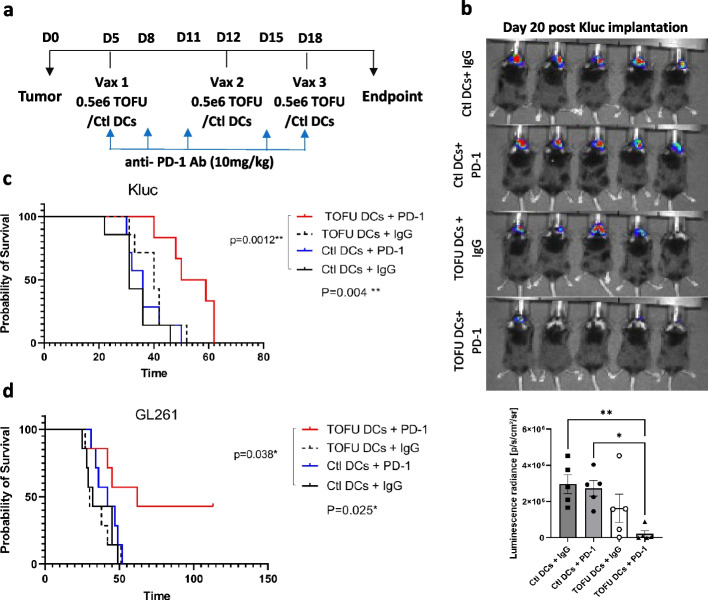
Therapeutic efficacy of TOFU mRNA vaccines in combination with ICI. **a** Timeline of therapy administration for the TOFU vaccine plus anti-PD-1 Ab combination approach. **b** Kluc tumor growth measurement using in vivo luminescence imaging at 48 h after the third vaccine treatment on day 20 following the tumor implantation (*n* = 5 per group). Statistical analysis was done using one-way ANOVA and Tukey’s multiple comparisons; *p* < 0.05 is *, and *p* < 0.01 is **. **c** Survival curve of Kluc tumor-bearing mice treated with Kluc TOFU mRNA vaccine plus anti-PD-1 Ab (TOFU DC + PD-1) and control treatments (Ctl DCs + IgG, Ctl DCs + PD-1, and TOFU DCs + IgG) (*n* = 6 to 7). **d** Survival curve of GL261 tumor-bearing mice treated with GL261 TOFU DCs + PD1 combination and control treatments (*n* = 7). Statistical analysis was performed using the log-rank (Mantel-Cox) test with significance at *p* < 0.05

We next investigated the anti-tumor efficacy of the TOFU mRNA vaccines. Given the immunosuppressive nature of GBM tumors and their inter and intra-tumoral heterogeneity, a combinatorial therapeutic approach was applied. Increased PD-L1 expression, which functions as an immune checkpoint for antigen-specific T-cells, has been observed in GBM tumors in response to inflammation at the tumor site [8, 29, 54]. Therefore, we investigated the anti-tumor efficacy of the TOFU mRNA vaccines in combination with anti-PD-1 ICI. GBM tumor-bearing mice were treated with three weekly doses of TOFU-DC vaccines and anti-PD-1 antibody (Ab) (TOFU-DCs + PD-1) (Fig. 3a), with unloaded control DCs (or Ctl-DCs) and isotype IgG Ab as controls (treatment groups: Ctl-DCs + IgG, Ctl-DCs + PD-1, and TOFU-DCs + IgG). The TOFU-DCs + PD-1 treatment was significantly more effective in slowing Kluc tumor progression compared to the Ctl DCs + IgG and Ctl DCs + PD-1 treatments as measured by bioluminescence intensity (Fig. 3b). The TOFU-DCs + IgG treatment group also showed a decrease in tumor progression; however, it was not significant. Improved survival benefit was observed in the TOFU-DCs + PD-1 combination group with a median survival of 54.5 days, compared to 31 days in the Ctl-DCs + IgG group, 36 days in the Ctl-DCs + PD-1 group, and 40 days in the TOFU-DCs + IgG treatment group (Fig. 3c).

We additionally evaluated the anti-tumor efficacy in the GL261 model and observed improved efficacy in inhibiting tumor growth (Additional file 1: Fig. S3a). Increased survival benefit was observed in tumor-bearing mice treated with TOFU-DCs + PD-1 combination with a median survival of 62 days as compared to 32 days in the Ctl-DCs + IgG group, 42 days in the Ctl-DCs + PD-1 group, and 30 days in the TOFU-DCs + IgG treatment group (Fig. 3d). Interestingly, re-challenge of the long-term survivors with GL261 resulted in complete tumor rejection, indicating a durable memory response to the tumor antigens (Additional file 1: Fig. S3b). Our data demonstrates a synergistic effect of the vaccines with anti-PD-1, possibly by addressing immunosuppression and poor immunogenicity of the tumor, and improves the outcome in GBM hosts over monotherapy alone [55].

### Reprograming of the tumor microenvironment following TOFU mRNA vaccines and ICI treatment

**Fig. 4:**
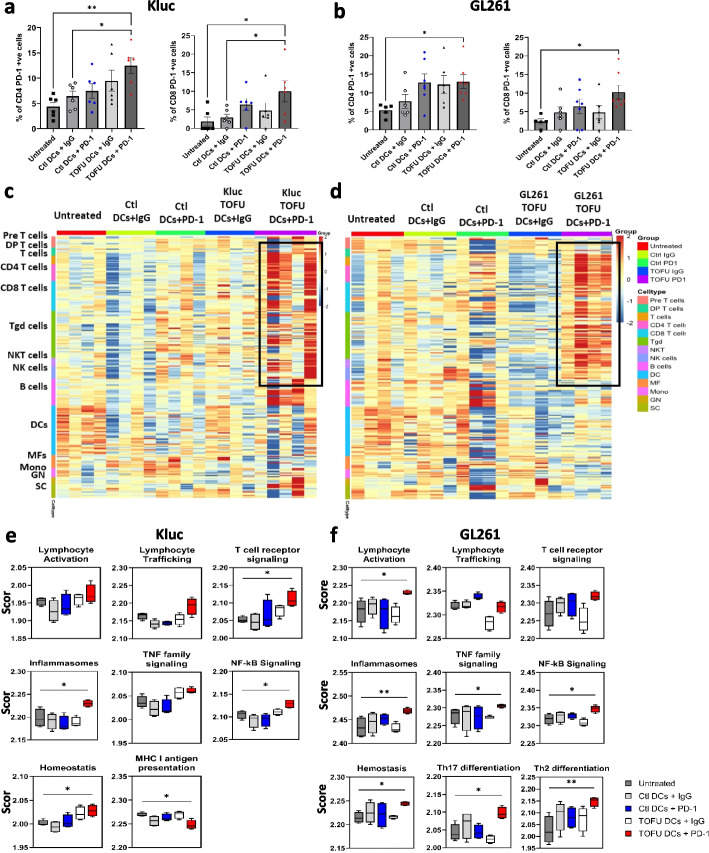
Reprograming of the tumor microenvironment following treatment with TOFU mRNA vaccines in combination with ICI. **a**–**b** Flow cytometry analysis of PD-1 expression on CD4 + and CD8 + T-cells in the peripheral blood of TOFU DCs + PD-1 or control-treated Kluc (**a**) or GL261 (**b**) tumor-bearing mice (*n* = 5 to 6 per group). Statistical analysis was done using one-way ANOVA and Dunnett’s multiple comparisons, *p* < 0.05. **c**–**d** Heatmap showing immune-cell deconvolution from the RNA-seq data of isolated CD45 + ve immune cells from the Kluc (**c**) and GL261 (**d**) tumors following treatment (*n* = 4 to 5). **e**–**f** Pathway-based gene expression analysis in the immune cells for Kluc (**e**) and the GL261 (**f**) tumors using the nCounter Immunology Panel from NanoString (*n* = 4 to 5). Statistical analysis was performed using Kruskal–Wallis and Dunn’s multiple comparison tests for TOFU DC + PD-1 to untreated mice comparison; *p* < 0.05

Analysis of peripheral T-cells using flow cytometry revealed an increase in PD-1 expression on CD4 + and CD8 + T-cells in TOFU-DCs + PD-1 treated Kluc (Fig. 4a) and GL261 (Fig. 4b) tumor-bearing mice supporting systemic activation of T-cells as previously described [56–58]. The PD-1 expression on CD4 + T-cells was also higher in the Ctl-DCs + PD-1 and TOFU-DCs + IgG groups, however, it was not significant. We next assessed the immune response within the tumor microenvironment (TME). GBM tumors are widely considered to be “immunologically cold”, meaning the TME is largely composed of myeloid-derived suppressor cells and macrophages with limited lymphocyte infiltration [59]. To evaluate the immune cell phenotypes in the TME and the involvement of key immune cell activation and response pathways, we performed bulk RNA-seq on CD45 + cells isolated from TME [39, 40]. Results from immune cell deconvolution of the gene expression data showed upregulation of lymphocytes, including CD4 + and CD8 + T-cells, γδ T-cells, NK cells, and NKT cells and a relative downregulation of myeloid cell signature including macrophages, monocytes, and DCs in the TOFU-DCs + PD-1 treated tumors in both Kluc (Fig. 4c) and GL261 (Fig. 4d) tumors and in B-cell gene signature in the Kluc tumors (Fig. 4c). In comparison, the Ctl-DCs + PD-1 and TOFU-DCs + IgG groups showed modest gene expression signature of the lymphocyte populations, while untreated and Ctl-DCs + IgG groups showed a cold tumor microenvironment in the Kluc model. All control groups, including the Ctl-DCs + PD-1 and TOFU-DCs + IgG groups, showed a cold tumor microenvironment in the GL261 model.

Immunology response pathway analysis showed lymphocyte activation, cytokine signaling, inflammasomes, Th17 differentiation, Th2 differentiation, NF-kB signaling, and TNF family signaling as significantly upregulated pathways in the TOFU-DCs + PD-1 treated GL261 tumor-bearing mice compared to untreated (Fig. 4f). In the more invasive and less immunogenic Kluc tumors, we observed a significant upregulation in the T-cell receptor signaling, inflammasomes, and NF-κB signaling pathways, with trends for increase in lymphocyte activation, lymphocyte trafficking, and TNF family signaling following the TOFU-DCs + PD-1 treatment (Fig. 4e). Interestingly, downregulation in MHC I antigen presentation on immune cells was observed in the TOFU-DCs + PD-1 treated Kluc mice. Together these findings demonstrate an increase in lymphocyte infiltration and immune cell response indicating conversion of GBM tumors from immunologically cold to hot in both the Kluc and GL261 tumors.

### Therapeutic efficacy of TOFU mRNA vaccines in combination with ACT

We have previously established an ACT platform that employs mRNA-pulsed DCs to ex vivo expand tumor antigen-specific T-cells, which are then adoptively transferred with hematopoietic stem cells (HSCs) to tumor-bearing hosts following 9 Gy total-body irradiation (TBI) [17, 19]. We first evaluated the TOFU antigen-reactive T-cells expanded ex vivo with TOFU-DCs for their capacity to recognize GBM tumor cells and APCs electroporated with TOFU mRNA*.* Higher secretion of IFN-γ was detected in Kluc TOFU antigen-specific T cells co-cultured with Kluc tumor cells and Kluc TOFU mRNA compared to non-specific GFP RNA (Additional file 1: Fig. S4a). Furthermore, TOFU antigen-specific T-cells demonstrate effective killing of Kluc and GL261 cells (Additional file 1: Fig. S4b–c) following co-culture with the tumor cells at various effector: target cell ratios.

**Fig. 5:**
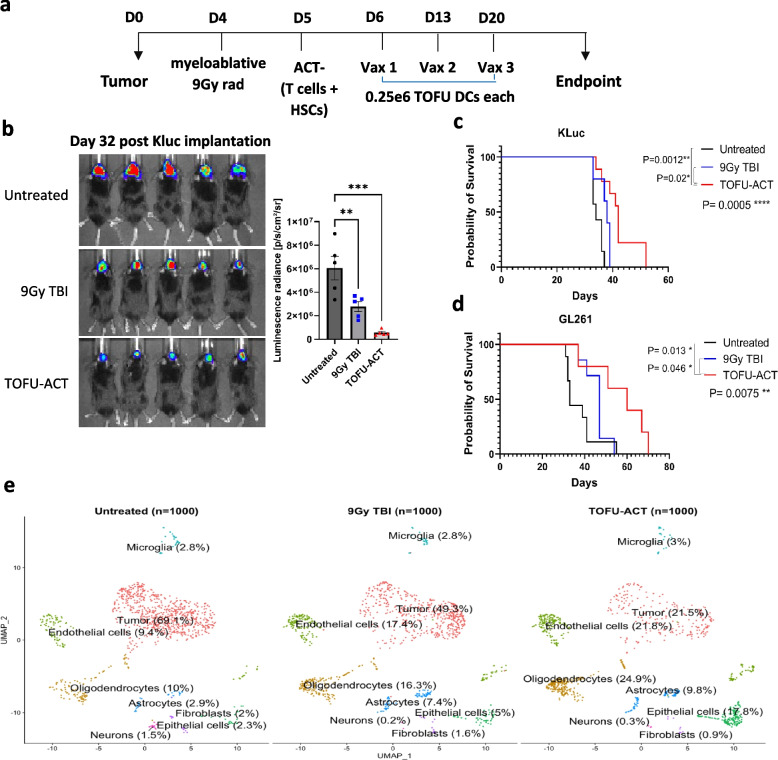
Therapeutic efficacy of TOFU mRNA vaccines in combination with ACT. **a** Timeline for therapy administration for the TOFU vaccine plus ACT combination approach following host conditioning with 9 Gy TBI and HSCs. **b** Kluc tumor growth measurement using in vivo luminescence imaging at day 32 after the tumor implantation (*n* = 5 per group). Statistical analysis was done using one-way ANOVA and Tukey’s multiple comparisons; *p* < 0.05 is *, *p* < 0.01 is **, and *p* < 0.001 is ***. **c** Survival curve of Kluc tumor-bearing mice treated with Kluc TOFU-ACT and control treatments (*n* = 7 to 9). **d** Survival curve of GL261 tumor-bearing mice treated with GL261 TOFU-ACT and control treatments (*n* = 7 to 9). Statistical analysis was performed using the log-rank (Mantel-Cox) test with significance at *p* < 0.05. **e** Uniform Manifold Approximation and Projection (UMAP) of 1000 CD45 − ve single cells from the TOFU-ACT and control groups which were resolved into 8 clusters. The cell type and the percentage of cells are labeled across the respective population

We next incorporated the ex vivo expanded TOFU antigen-reactive T-cells into the experimental design of ACT (TOFU-ACT group), where untreated mice (Untreated group) and mice receiving 9 Gy TBI plus HSC rescue without ACT (9 Gy TBI group) served as controls (Fig. 5a). The antitumor immunity in the TOFU-ACT group was further maintained with weekly mRNA-pulsed DC vaccines. The TOFU-ACT treatment significantly delayed tumor progression as measured by bioluminescence intensity compared to untreated mice (Fig. 5b) and significantly improved survival in Kluc tumor-bearing mice (treatment median survival of 42 days compared to 34 days in untreated mice) (Fig. 5c). The TOFU-ACT combination therapy also resulted in a significant improvement in survival in the GL261 tumor-bearing mice (treatment median survival of 60 days compared to 33 days in untreated mice) (Fig. 5d).

### Reprograming of the tumor microenvironment following treatment with TOFU-ACT

**Fig. 6:**
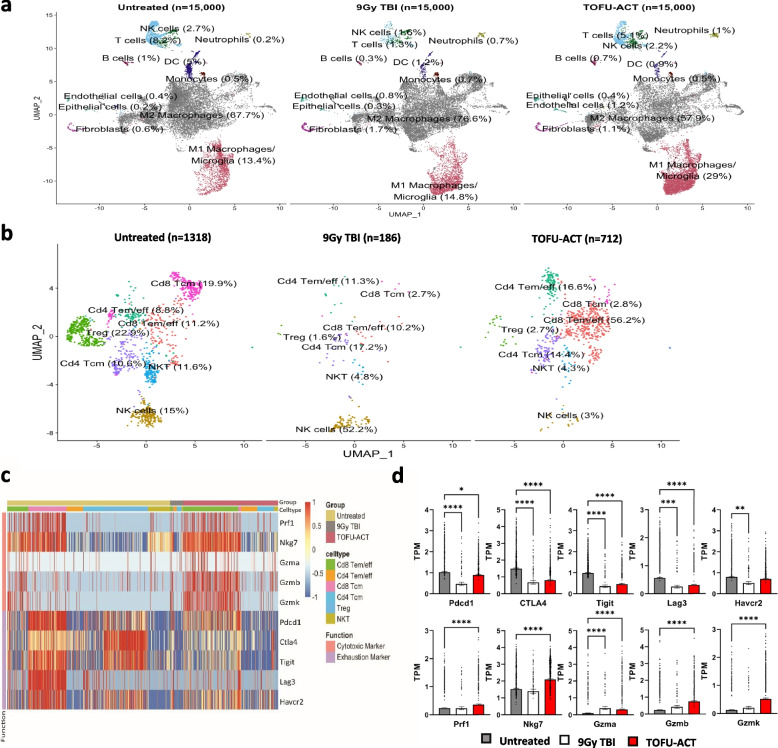
Reprogramming of the tumor microenvironment following treatment with TOFU-ACT. **a** UMAP of 15,000 CD45 + ve single cells from the TOFU-ACT and control groups which were resolved into 11 major clusters. The cell type and the percentage of cells are labeled across the respective population. **b** UMAP of T-cells and NK single cells from the TOFU-ACT and control groups which were resolved into 7 major clusters. The cell type and the percentage of cells are labeled across the respective population. **c**–**d** Analysis of gene expression of common T-cell exhaustion and cytotoxic markers in the three treatment groups across the different T-cell populations. The heatmap of gene expression is shown in **c** and the quantification of the same is shown in (**d**). Statistical analysis was performed using Kruskal–Wallis and Dunn’s test for multiple comparisons; p < 0.05 is *, *p* < 0.01 is **, *p* < 0.001 is ***, and *p* < 0.0001 is ****

To understand the intra-tumoral immune cell response induced by ACT employing TOFU mRNA encoded antigens, and identify potential interventions that could overcome treatment resistance, we performed single cell (sc) RNA-seq on CD45 + immune cells and CD45 − tumor and normal brain cells that were isolated from the tumors of mice treated with TOFU-ACT or control treatments (Fig. 6a) [44]. Using Uniform Manifold Approximation and Projection (UMAP), the differential gene expression from the scRNA-seq data resolved the CD45 − cells into eight major clusters, including tumor cells, oligodendrocytes, astrocytes, neurons, fibroblasts, epithelial cells, endothelial cells, and microglia (Fig. 5e). Consistent with the survival data, we observed a marked reduction in tumor cell population in the TOFU-ACT treated mice (21.5%) as compared to the untreated (69%) and 9 Gy TBI treated mice (49.3%) (Fig. 5e). Pan-cancer pathway analysis of the tumor cells revealed downregulation to the immune response in the adaptive, innate, humoral, antigen processing, inflammation, interleukins, cytokine and chemokine receptors, and interferon pathways in the TOFU-ACT treated mice compared to untreated (Additional file 1: Fig. S5a–b). We also observed an increase in the cancer progression and a decrease in senescence pathways, but no changes in apoptosis, cell cycle, or adhesion. These inherent or treatment-acquired properties of residual tumor cells offer insights into mechanisms of immune resistance and escape.

We next conducted a comprehensive analysis of CD45 + single cells in the tumors, obtaining scRNA-seq data from 15,000 single cells per group. The CD45 + immune cells were resolved into 11 major clusters including M1 macrophages, M2 macrophages, T-cells, NK cells, B-cells, DCs, monocytes, neutrophils, and some contamination from endothelial cells, epithelial cells, and fibroblasts (Fig. 6a). Microglia specific gene signature was found to overlap with M1 macrophage cluster. We found an increase in M1 macrophages (untreated-13.4%, 9 Gy TBI-14.8%, and TOFU-ACT-29%) and a corresponding decrease in M2 macrophages (untreated-67.7%, 9 Gy TBI-76.6%, and TOFU-ACT-57.9%) in the TOFU-ACT treated mice compared to controls, suggesting a more pro-inflammatory tumor microenvironment [60] (Fig. 6a).

We further resolved the NK cell/T-cell population into 7 clusters including CD8 + T central memory cells (Cd8 Tcm), CD8 + T effector memory/effector cells (Cd8 Tem/eff), CD4 + T central memory cells (Cd4 Tcm), CD4 + T effector memory/effector cells (Cd4 Tem/eff), Treg cells, NK cells, and NKT cells (Fig. 6b). Results showed an increase in Cd8 Tem/eff T-cells (untreated-11.2%, 9 Gy TBI-10.2%, and TOFU-ACT-56.2%) and Cd4 Tem/eff (untreated-8.8%, 9 Gy TBI-11.3%, and TOFU-ACT-16.6%), and a decrease in Cd8 Tcm cells (untreated-19.9%, 9 Gy TBI-2.7%, and TOFU-ACT-2.8%) in TOFU-ACT treated mice compared to controls. We also observed a decrease in Tregs (untreated-22.9%, 9 Gy TBI-1.6%, and TOFU-ACT-2.7%), NKT cells (untreated-11.6%, 9 Gy TBI-4.8%, and TOFU-ACT-4.3%), and NK cells (untreated-15%, 9 Gy TBI-52.2%, and TOFU-ACT-3%) in mice treated with TOFU-ACT. These findings indicate a significant shift towards effector/effector-memory T-cells in the tumor microenvironment of TOFU-ACT treated animals.

Interestingly, the increase in effector/effector memory CD4 + and CD8 + T-cells and the decrease in central memory T-cells was also observed in peripheral T cells following TOFU-ACT treatment (Additional file 1: Fig. S6a–b). Additionally, an increase in the expression of PD-1 was observed on CD4 + and CD8 + peripheral T-cells in the TOFU-ACT-treated mice compared to untreated, suggesting systemic activation (Additional file 1: Fig. S6a–b).

### Determination of the effector function of T-cells following treatment with TOFU-ACT

We next evaluated the gene expression of several key cytotoxicity and exhaustion markers on T-cells [26] (Fig. 6c) and found significantly decreased expression of the exhaustion markers *Pdcd1*,* CTLA4*,* Tigit*, and *Lag3* in the TOFU-ACT treated mice compared to untreated (Fig. 6c–d). A reduction in the expression of Tim3 (gene name *Havcr2*) was also observed in the mice treated with TOFU-ACT, however, it was not significant. Conversely, the expression of the cytotoxicity markers *Prf1*,* Nkg7*,* Gzma*,* Gzmb*, and *Gzmk* was significantly upregulated in the TOFU-ACT-treated mice (Fig. 6c–d). We observed the expression of cytotoxicity and exhaustion markers mainly on CD8 + T-cells in all the groups, with limited expression on CD4 + T-cells (Fig. 6c). An exception was noted in T-reg cells, which showed upregulation of the exhaustion markers *Pdcd1*,* CTLA4*,* Tigit*, and *Havcr2*, particularly in untreated mice (Fig. 6c).

We next examined the co-expression of inhibitory molecules on CD4 + and CD8 + T-cells to evaluate their degree of exhaustion [26]. The number of CD8 + and CD4 + T-cells that co-expressed all five exhaustion markers was markedly lower in the TOFU-ACT treated mice (22% and 3.3%) compared to untreated mice (56% and 12%) and 9 Gy TBI (33% and 3.8%) (Additional file 1: Fig. S7a–b). To identify which subset of T-cells concomitantly expressed exhaustion markers, we examined UMAP analysis of *Pdcd1*,* Lag3*, and* Tim3* markers on lymphocytes (as shown in Fig. 6b) and found that Cd8 and Cd4 Tcm but not the Tem/eff cell populations displayed a triple positive phenotype (Additional file 1: Fig. S7c). Enhanced T-cell cytotoxicity and decreased exhaustion were further corroborated by immune-exhaustion pathway-based gene expression analysis (Additional file 1: Fig. S7d–e).

We evaluated the interactome data between the different cell populations in the tumor microenvironment (Additional file 1: Fig. S8a). We observed increased cell–cell interactions of CD8 Tcm cells and decreased interactions of CD4 Tem/eff cells with macrophages, tumor cells, and DCs in the TOFU-ACT treated mice compared to untreated (Additional file 1: Fig. S8b). The decreased interactions with CD4 Tem/eff may likely be due to decrease in MHC-II interactions in the TOFU-ACT-treated mice compared to untreated (Additional file 1: Fig. S8c). The interactome data showed significantly lower interactions between inhibitory receptor-ligands such as PD-L1, PD-L2, TIGIT, and Tim-3 in the TOFU-ACT treated mice compared to untreated (Additional file 1: Fig. S8d). Interestingly, co-stimulatory interactions with CD86 and CD80 which are involved in T-cell activation were also downregulated in the TOFU-ACT-treated mice (Additional file 1: Fig. S8d). Among interactions that were upregulated in the TOFU-ACT treated mice compared to untreated was inducible co-stimulatory molecule (ICOS) which is involved in T-cell activation [61], pleiotrophin (PTN) which is implicated in migration and T-cell proliferation [62], CD39 which is expressed by activated T-cells [63], IL4 which is a potent regulator of immunity and T-cell differentiation [64], a proliferation-inducing ligand (APRIL) which acts as a co-stimulator for T-cell proliferation [65], intracellular adhesion molecule (ICAM) which is involved in T-cell migration and activation [66], and fibronectin 1 (FN1) which is implicated in T-cell proliferation following interaction with the extracellular environment [67] (Additional file 1: Fig. S8d). Additional interactions upregulated in the TOFU-ACT treated mice are involved in angiogenesis and extracellular matrix proteins such as periostin, angiopoietin (ANGPT), vascular endothelial growth factor (VEGF), collagen, and laminin [68, 69].

### T-cell receptor repertoire analysis following treatment with TOFU-ACT

**Fig. 7:**
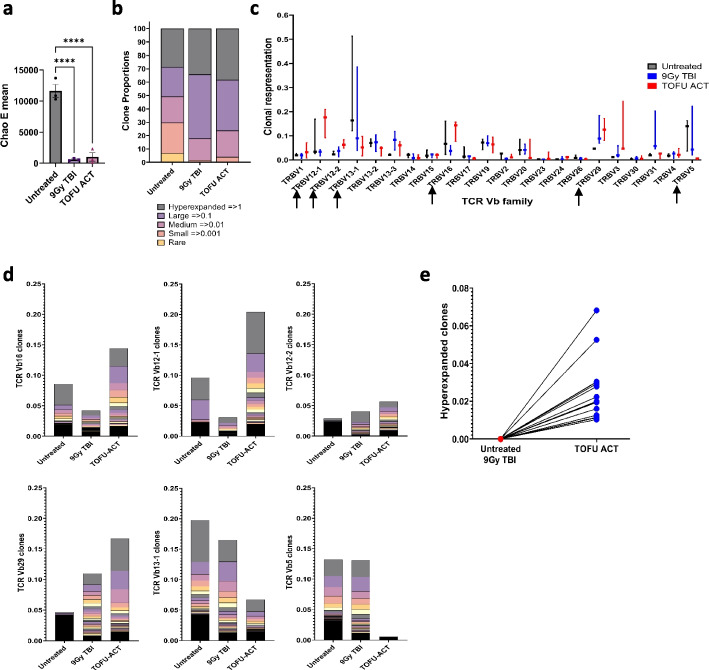
T-cell receptor sequencing following the TOFU-ACT treatment. **a** Chao E mean score analysis for determining the clonal diversity of T-cells (*n* = 3 per group). Statistical analysis was performed using Kruskal–Wallis and Dunn’s test for multiple comparisons; *p* < 0.05 is *, *p* < 0.01 is **, *p* < 0.001 is ***, and *p* < 0.0001 is ****. **b** T-cell receptor repertoire and clonal expansion using TCR-seq analysis (*n* = 3 per group). The distribution of clones is shown as hyperexpanded, large, medium, small, and rare based on the expansion of clones and proportion out of the total number of reads. **c** The expression of TCR Vβ families in the T-cell repertoire of all three treatment groups following therapy. Black arrows show the Vβ families which are either upregulated or downregulated in the TOFU-ACT-treated tumors compared to controls. **d** The proportion of individual T-cell clones within different TCR Vβ families. **e** The expression of the individual hyperexpanded clones in the TOFU-ACT treated tumors compared to the untreated and 9 Gy TBI treated tumors

We conducted TCR-seq to compare the diversity, clonal expansion, and T-cell receptor (TCR) repertoire in mice treated with TOFU-ACT versus controls [41]. We observed lower T-cell diversity following TOFU-ACT or 9 Gy TBI treatment compared to untreated mice as determined using the Chao E mean value, most likely caused due to the lymphodepletion (Fig. 7a). Clones were further classified based on their number of reads as hyperexpanded (> = 1% of the total), large (> = 0.1%), medium (> = 0.01%), small (> = 0.001%), and rare. In mice treated with TOFU-ACT and 9 Gy TBI, greater than 95% of reads were represented by hyperexpanded, large, or medium clones and < 5% were represented by small or rare clones as compared to 70% and 30% in the untreated mice respectively, indicating clonal expansion following treatment (Fig. 7b).

Despite similarities in diversity and clonal expansion trend between the TOFU-ACT and 9 Gy TBI control, the TCR repertoire and Vβ family distribution demonstrated major differences (Fig. 7c). The TOFU-ACT treated mice had increased expansion of T-cell clones in the Vβ12-1, Vβ12-2, Vβ16, and Vβ29 families, and decreased expansion in the Vβ13-1 and Vβ5 families compared to the 9 Gy TBI and untreated mice (Fig. 7c–d). Moreover, the 15 hyperexpanded clones observed in the TOFU-ACT treated mice were absent in the untreated mice and 9 Gy TBI treated mice (Fig. 7e). These results demonstrate that the changes in the TCR repertoire, Vβ family distribution, and hyper-expanded clones were in response to the TOFU-ACT treatment.

## Discussion

To date, cancer mRNA vaccines have been evaluated in several clinical trials and have demonstrated promising early results [16, 18, 70, 71]. mRNA cancer vaccines may address the urgency of developing promising treatment modalities for other refractory solid tumors, including GBM and MB. However, tumors such as GBM typically have a low mutation burden and the heterogeneous expression of antigens throughout the tumor presents a major challenge in effectively eliminating all cancer cells and preventing the potential resurgence of tumor cells that lack the targeted antigens. These challenges highlight the need for multi-antigen targeting combinations to expand the effectiveness of immunotherapy. Here, we have established a personalized and customizable mRNA vaccine platform which leverages a novel gene enrichment strategy to target multiple tumor antigens identified using our cancer immunogenomics pipeline, in a single vaccine. Interestingly, some of the immunogenic TAAs encoded for isoform-specific transcripts or genes found in the normal-tissue database but cross the expression threshold in the TME, making them prospective targets for T-cells or Ab-mediated therapies. Here, we demonstrate the capacity to generate tumor antigen-specific TOFU mRNA vaccines with greater than 85% enrichment efficiency and validate their immunogenicity in preclinical models of GBM and MB. Anti-tumor efficacy of our approach using combination immunotherapies was shown in two distinct preclinical models of GBM- the first model (Kluc) has a low mutational burden and is highly resistant to treatment, while the second model (GL261) is characterized by a high mutational burden. This allowed us to demonstrate the versatility of our platform and its ability to target tumors with a wide range of antigen burden.

There is an increasing awareness in the scientific community to incorporate immune monitoring strategies in both clinical and preclinical studies while evaluating treatment outcomes to identify interventions that enhance anti-tumor immunity. To gain a thorough understanding of the immune response elicited by our TOFU mRNA vaccine platform, we performed scRNA-seq and bulk-RNA seq that enabled high-resolution mapping of cellular heterogeneity and activation states in immune cells. Furthermore, we incorporated TCR sequencing to expand our understating of T-cell immunity and monitor changes in the tumor microenvironment following antigen exposure. In mice that received the TOFU DCs + PD-1 treatment, we show increased lymphocyte presence in the tumors, demonstrating the conversion of GBM tumors from immunologically cold to hot. Furthermore, our findings demonstrate a robust increase in effector/effector memory CD4 + and CD8 + T-cells and cytotoxicity markers in mice treated with TOFU-ACT. Although the expression of exhaustion markers was detected on T-cells in the TOFU-ACT-treated mice, their individual and concomitant expression was markedly lower compared to the untreated mice. Together our findings substantiate a robust increase in tumor-infiltrating lymphocytes characterized by enhanced effector function both intratumorally and systemically after TOFU mRNA-directed immunotherapies, resulting in favorable anti-tumor efficacy in the treated mice.

As a future direction, we aim to improve the efficacy of our TOFU-ACT platform by promoting memory T-cell responses and enhancing the involvement of CD4 + helper T-cells. The expression of exhaustion markers on the T-cells suggests that overcoming immune cell exhaustion by ICI, during the T-cell expansion, and after the ACT administration could further improve this strategy [72, 73]. The integration of cutting-edge technologies in vaccine development and immune monitoring will enhance the efficacy of these immunotherapies and remain an important area of investigation for cancer treatment strategies.

## Conclusions

Our mRNA-based immunotherapeutic approach of targeting a plurality of tumor antigens uniquely addresses the challenge of dealing with tumor antigenic heterogeneity and confronting the reality of patient-to-patient variation in antigen expression in the development of antigen-directed strategies. Our findings indicate that the use of mRNA-based antigen-directed immunotherapy leads to improved survival in GBM hosts by stimulating T-cell activation and effector function and by altering the immunosuppressive tumor microenvironment as determined using immune-monitoring techniques. These advancements in the field of mRNA therapeutics allow for more personalized and effective treatments for patients, thereby augmenting the role of immunotherapy as a powerful tool in the treatment of aggressive cancers.

### Supplementary Information

**Additional file 1. **This file includes all the supplementary figures and tables described in this manuscript.

**Additional file 2.** This extended file contains the names and number of antigens identified for human GBM tumors obtained from the TCGA database.

**Additional file 3.** This extended file contains the peptide information used for the immunogenicity assay for the 4 brain tumor models discussed in this paper.

## Data Availability

Raw sequencing data and processed datasets are available from NCBI GEO (BioProject: *PRJNA1054323*) *GSE251798, GSE251799*,* GSE251800*, and* GSE252247* for the GL261 DCs + PD-1 bulk RNA-seq, KR158 DCs + PD-1 bulk RNA-seq, KR158 TOFU ACT TCR-seq, and KR158 TOFU ACT scRNA-seq experiments respectively [39–41, 44]. The script for the O.R.A.N pipeline is being reviewed for licensing purposes at the University of Florida and is therefore not publicly available. However, it can be made available upon reasonable request sent to the corresponding author at Duane.Mitchell@neurosurgery.ufl.edu. Response time will typically be within 48 h. The scripts will be available after completing a Data Transfer Agreement with the Division of Sponsored Programs at UF Research.

## References

[1] Rosenberg SA, Dudley ME (2009). Adoptive cell therapy for the treatment of patients with metastatic melanoma. Curr Opin Immunol.

[2] Rosenberg SA, Restifo NP (2015). Adoptive cell transfer as personalized immunotherapy for human cancer. Science.

[3] Yang JC, Rosenberg SA (2016). Adoptive T-Cell Therapy for Cancer. Adv Immunol.

[4] Chen S, Zhang Z, Zheng X, Tao H, Zhang S, Ma J, Liu Z, Wang J, Qian Y, Cui P (2021). Response efficacy of PD-1 and PD-L1 inhibitors in clinical trials: a systematic review and meta-analysis. Front Oncol.

[5] Sun L, Zhang L, Yu J, Zhang Y, Pang X, Ma C, Shen M, Ruan S, Wasan HS, Qiu S (2020). Clinical efficacy and safety of anti-PD-1/PD-L1 inhibitors for the treatment of advanced or metastatic cancer: a systematic review and meta-analysis. Sci Rep.

[6] Akhavan D, Alizadeh D, Wang D, Weist MR, Shepphird JK, Brown CE (2019). CAR T cells for brain tumors: Lessons learned and road ahead. Immunol Rev.

[7] Filley AC, Henriquez M, Dey M (2017). Recurrent glioma clinical trial, CheckMate-143: the game is not over yet. Oncotarget.

[8] Hodges TR, Ott M, Xiu J, Gatalica Z, Swensen J, Zhou S, Huse JT, de Groot J, Li S, Overwijk WW (2017). Mutational burden, immune checkpoint expression, and mismatch repair in glioma: implications for immune checkpoint immunotherapy. Neuro Oncol.

[9] O'Rourke DM, Nasrallah MP, Desai A, Melenhorst JJ, Mansfield K, Morrissette JJD, Martinez-Lage M, Brem S, Maloney E, Shen A (2017). A single dose of peripherally infused EGFRvIII-directed CAR T cells mediates antigen loss and induces adaptive resistance in patients with recurrent glioblastoma. Sci Transl Med.

[10] Razavi SM, Lee KE, Jin BE, Aujla PS, Gholamin S, Li G (2016). Immune Evasion Strategies of Glioblastoma. Front Surg.

[11] Lee AH, Sun L, Mochizuki AY, Reynoso JG, Orpilla J, Chow F, Kienzler JC, Everson RG, Nathanson DA, Bensinger SJ (2021). Neoadjuvant PD-1 blockade induces T cell and cDC1 activation but fails to overcome the immunosuppressive tumor associated macrophages in recurrent glioblastoma. Nat Commun.

[12] Anderson EJ, Rouphael NG, Widge AT, Jackson LA, Roberts PC, Makhene M, Chappell JD, Denison MR, Stevens LJ, Pruijssers AJ (2020). Safety and immunogenicity of SARS-CoV-2 mRNA-1273 vaccine in older adults. N Engl J Med.

[13] Lorentzen CL, Haanen JB, Met O, Svane IM (2022). Clinical advances and ongoing trials on mRNA vaccines for cancer treatment. Lancet Oncol.

[14] Melnick K, Dastmalchi F, Mitchell D, Rahman M, Sayour EJ (2022). Contemporary RNA Therapeutics for Glioblastoma. Neuromolecular Med.

[15] Sahin U, Muik A, Derhovanessian E, Vogler I, Kranz LM, Vormehr M, Baum A, Pascal K, Quandt J, Maurus D (2020). COVID-19 vaccine BNT162b1 elicits human antibody and T(H)1 T cell responses. Nature.

[16] Batich KA, Reap EA, Archer GE, Sanchez-Perez L, Nair SK, Schmittling RJ, Norberg P, Xie W, Herndon JE, Healy P (2017). Long-term Survival in Glioblastoma with Cytomegalovirus pp65-Targeted Vaccination. Clin Cancer Res.

[17] Flores C, Pham C, Snyder D, Yang S, Sanchez-Perez L, Sayour E, Cui X, Kemeny H, Friedman H, Bigner DD (2015). Novel role of hematopoietic stem cells in immunologic rejection of malignant gliomas. Oncoimmunology.

[18] Mitchell DA, Batich KA, Gunn MD, Huang MN, Sanchez-Perez L, Nair SK, Congdon KL, Reap EA, Archer GE, Desjardins A (2015). Tetanus toxoid and CCL3 improve dendritic cell vaccines in mice and glioblastoma patients. Nature.

[19] Wildes TJ, Grippin A, Dyson KA, Wummer BM, Damiani DJ, Abraham RS, Flores CT, Mitchell DA (2018). Cross-talk between T Cells and Hematopoietic Stem Cells during Adoptive Cellular Therapy for Malignant Glioma. Clin Cancer Res.

[20] Grippin AJ, Wummer B, Wildes T, Dyson K, Trivedi V, Yang C, Sebastian M, Mendez-Gomez HR, Padala S, Grubb M (2019). Dendritic Cell-Activating Magnetic Nanoparticles Enable Early Prediction of Antitumor Response with Magnetic Resonance Imaging. ACS Nano.

[21] Frederico SC, Hancock JC, Brettschneider EES, Ratnam NM, Gilbert MR, Terabe M (2021). Making a cold tumor hot: the role of vaccines in the treatment of glioblastoma. Front Oncol.

[22] Weller M, Roth P, Preusser M, Wick W, Reardon DA, Platten M, Sampson JH (2017). Vaccine-based immunotherapeutic approaches to gliomas and beyond. Nat Rev Neurol.

[23] Miao L, Zhang Y, Huang L (2021). mRNA vaccine for cancer immunotherapy. Mol Cancer.

[24] Bonehill A, Heirman C, Tuyaerts S, Michiels A, Breckpot K, Brasseur F, Zhang Y, Van Der Bruggen P, Thielemans K (2004). Messenger RNA-electroporated dendritic cells presenting MAGE-A3 simultaneously in HLA class I and class II molecules. J Immunol.

[25] Hilf N, Kuttruff-Coqui S, Frenzel K, Bukur V, Stevanovic S, Gouttefangeas C, Platten M, Tabatabai G, Dutoit V, van der Burg SH (2019). Actively personalized vaccination trial for newly diagnosed glioblastoma. Nature.

[26] Keskin DB, Anandappa AJ, Sun J, Tirosh I, Mathewson ND, Li S, Oliveira G, Giobbie-Hurder A, Felt K, Gjini E (2019). Neoantigen vaccine generates intratumoral T cell responses in phase Ib glioblastoma trial. Nature.

[27] Wu C, Qin C, Long W, Wang X, Xiao K, Liu Q (2022). Tumor antigens and immune subtypes of glioblastoma: the fundamentals of mRNA vaccine and individualized immunotherapy development. J Big Data.

[28] Reilly KM, Loisel DA, Bronson RT, McLaughlin ME, Jacks T (2000). Nf1;Trp53 mutant mice develop glioblastoma with evidence of strain-specific effects. Nat Genet.

[29] Pham CD, Flores C, Yang C, Pinheiro EM, Yearley JH, Sayour EJ, Pei Y, Moore C, McLendon RE, Huang J (2016). Differential immune microenvironments and response to immune checkpoint blockade among molecular subtypes of murine medulloblastoma. Clin Cancer Res.

[30] Li B, Dewey CN (2011). RSEM: accurate transcript quantification from RNA-Seq data with or without a reference genome. BMC Bioinformatics.

[31] McKenna A, Hanna M, Banks E, Sivachenko A, Cibulskis K, Kernytsky A, Garimella K, Altshuler D, Gabriel S, Daly M (2010). The Genome Analysis Toolkit: a MapReduce framework for analyzing next-generation DNA sequencing data. Genome Res.

[32] Hundal J, Carreno BM, Petti AA, Linette GP, Griffith OL, Mardis ER, Griffith M (2016). pVAC-Seq: A genome-guided in silico approach to identifying tumor neoantigens. Genome Med.

[33] Hundal J, Kiwala S, McMichael J, Miller CA, Xia H, Wollam AT, Liu CJ, Zhao S, Feng YY, Graubert AP (2020). pVACtools: a computational toolkit to identify and visualize cancer neoantigens. Cancer Immunol Res.

[34] Ellrott K, Bailey MH, Saksena G, Covington KR, Kandoth C, Stewart C, Hess J, Ma S, Chiotti KE, McLellan M, Sofia HJ (2018). Scalable open science approach for mutation calling of tumor exomes using multiple genomic pipelines. Cell Syst.

[35] Szolek A, Schubert B, Mohr C, Sturm M, Feldhahn M, Kohlbacher O (2014). OptiType: precision HLA typing from next-generation sequencing data. Bioinformatics.

[36] Bai  Y, Wang  D, Fury  W (2018). PHLAT: inference of high-resolution HLA types from RNA and whole exome sequencing. HLA Typing: Methods and Protocols.

[37] Bolotin DA, Poslavsky S, Mitrophanov I, Shugay M, Mamedov IZ, Putintseva EV, Chudakov DM (2015). MiXCR: software for comprehensive adaptive immunity profiling. Nat Methods.

[38] Hanzelmann S, Castelo R, Guinney J (2013). GSVA: gene set variation analysis for microarray and RNA-seq data. BMC Bioinformatics.

[39] Trivedi V, Yang C, Klippel K, Yegorov O, von Roemeling C, Hoang-Minh L, Fenton G, Ogando-Rivas E, Castillo P, Moore G, Long-James K, Dyson K, Doonan B, Flores C, Mitchell DA. mRNA-based precision targeting of neoantigens and tumor-associated antigens in malignant brain tumors. NCBI GEO, GSE251798. 10.1186/s13073-024-01281-z PMC10809449 38268001

[40] Trivedi V, Yang C, Klippel K, Yegorov O, von Roemeling C, Hoang-Minh L, Fenton G, Ogando-Rivas E, Castillo P, Moore G, Long-James K, Dyson K, Doonan B, Flores C, Mitchell DA. mRNA-based precision targeting of neoantigens and tumor-associated antigens in malignant brain tumors. NCBI GEO, GSE251799. 10.1186/s13073-024-01281-z PMC10809449 38268001

[41] Trivedi V, Yang C, Klippel K, Yegorov O, von Roemeling C, Hoang-Minh L, Fenton G, Ogando-Rivas E, Castillo P, Moore G, Long-James K, Dyson K, Doonan B, Flores C, Mitchell DA.mRNA-based precision targeting of neoantigens and tumor-associated antigens in malignant brain tumors. NCBI GEO, GSE251800. 10.1186/s13073-024-01281-z PMC10809449 38268001

[42] Stoeckius M, Zheng S, Houck-Loomis B, Hao S, Yeung BZ, Mauck WM, Smibert P, Satija R (2018). Cell Hashing with barcoded antibodies enables multiplexing and doublet detection for single cell genomics. Genome Biol.

[43] Hao Y, Hao S, Andersen-Nissen E, Mauck WM 3rd, Zheng S, Butler A, Lee MJ, Wilk AJ, Darby C, Zager M, et al. Integrated analysis of multimodal single-cell data. Cell. 2021;184(13):3573–3587 e3529. 10.1016/j.cell.2021.04.048 PMC8238499 34062119

[44] Trivedi V, Yang C, Klippel K, Yegorov O, von Roemeling C, Hoang-Minh L, Fenton G, Ogando-Rivas E, Castillo P, Moore G, Long-James K, Dyson K, Doonan B, Flores C, Mitchell DA.mRNA-based precision targeting of neoantigens and tumor-associated antigens in malignant brain tumors. NCBI GEO, GSE252247. 10.1186/s13073-024-01281-z PMC10809449 38268001

[45] Aran D, Looney AP, Liu L, Wu E, Fong V, Hsu A, Chak S, Naikawadi RP, Wolters PJ, Abate AR (2019). Reference-based analysis of lung single-cell sequencing reveals a transitional profibrotic macrophage. Nat Immunol.

[46] Linderman GC, Zhao J, Roulis M, Bielecki P, Flavell RA, Nadler B, Kluger Y (2022). Zero-preserving imputation of single-cell RNA-seq data. Nat Commun.

[47] Aibar S, Gonzalez-Blas CB, Moerman T, Huynh-Thu VA, Imrichova H, Hulselmans G, Rambow F, Marine JC, Geurts P, Aerts J (2017). SCENIC: single-cell regulatory network inference and clustering. Nat Methods.

[48] Jin S, Guerrero-Juarez CF, Zhang L, Chang I, Ramos R, Kuan CH, Myung P, Plikus MV, Nie Q (2021). Inference and analysis of cell-cell communication using Cell Chat. Nat Commun.

[49] Blass E, Ott PA (2021). Advances in the development of personalized neoantigen-based therapeutic cancer vaccines. Nat Rev Clin Oncol.

[50] Rivero-Hinojosa S, Grant M, Panigrahi A, Zhang H, Caisova V, Bollard CM, Rood BR (2021). Proteogenomic discovery of neoantigens facilitates personalized multi-antigen targeted T cell immunotherapy for brain tumors. Nat Commun.

[51] Haen SP, Loffler MW, Rammensee HG, Brossart P (2020). Towards new horizons: characterization, classification and implications of the tumour antigenic repertoire. Nat Rev Clin Oncol.

[52] Cella M, Sallusto F, Lanzavecchia A (1997). Origin, maturation and antigen presenting function of dendritic cells. Curr Opin Immunol.

[53] Lanzavecchia A, Sallusto F (2001). Regulation of T cell immunity by dendritic cells. Cell.

[54] Reardon DA, Gokhale PC, Klein SR, Ligon KL, Rodig SJ, Ramkissoon SH, Jones KL, Conway AS, Liao X, Zhou J (2016). Glioblastoma eradication following immune checkpoint blockade in an orthotopic, immunocompetent model. Cancer Immunol Res.

[55] Antonios JP, Soto H, Everson RG, Orpilla J, Moughon D, Shin N, Sedighim S, Yong WH, Li G, Cloughesy TF (2016). PD-1 blockade enhances the vaccination-induced immune response in glioma. JCI Insight.

[56] Gros A, Tran E, Parkhurst MR, Ilyas S, Pasetto A, Groh EM, Robbins PF, Yossef R, Garcia-Garijo A, Fajardo CA (2019). Recognition of human gastrointestinal cancer neoantigens by circulating PD-1+ lymphocytes. J Clin Invest.

[57] Jing WQ, Gershan JA, Blitzer GC, Palen K, Weber J, McOlash L, Riese M, Johnson BD (2017). Adoptive cell therapy using PD-1(+) myeloma-reactive T cells eliminates established myeloma in mice. J Immunother Cancer.

[58] Kamphorst AO, Pillai RN, Yang S, Nasti TH, Akondy RS, Wieland A, Sica GL, Yu K, Koenig L, Patel NT (2017). Proliferation of PD-1+ CD8 T cells in peripheral blood after PD-1-targeted therapy in lung cancer patients. Proc Natl Acad Sci U S A.

[59] Habashy KJ, Mansour R, Moussalem C, Sawaya R, Massaad MJ (2022). Challenges in glioblastoma immunotherapy: mechanisms of resistance and therapeutic approaches to overcome them. Brit J Cancer.

[60] Liu JY, Geng XF, Hou JX, Wu GS (2021). New insights into M1/M2 macrophages: key modulators in cancer progression. Cancer Cell Int.

[61] Mahajan S, Cervera A, MacLeod M, Fillatreau S, Perona-Wright G, Meek S, Smith A, MacDonald A, Gray D (2007). The role of ICOS in the development of CD4 T cell help and the reactivation of memory T cells. Eur J Immunol.

[62] Sorrelle N, Dominguez ATA, Brekken RA (2017). From top to bottom: midkine and pleiotrophin as emerging players in immune regulation. J Leukocyte Biol.

[63] Kortekaas KE, Santegoets SJ, Sturm G, Ehsan I, van Egmond SL, Finotello F, Trajanoski Z, Welters MJP, van Poelgeest MIE, van der Burg SH (2020). CD39 Identifies the CD4(+) Tumor-Specific T-cell Population in Human Cancer. Cancer Immunol Res.

[64] Heeb LEM, Egholm C, Boyman O (2020). Evolution and function of interleukin-4 receptor signaling in adaptive immunity and neutrophils. Genes Immun.

[65] Stein JV, Lopez-Fraga M, Elustondo FA, Carvalho-Pinto CE, Rodriguez D, Gomez-Caro R, de Jong J, Martinez-A C, Medema JP, Hahne M (2002). APRIL modulates B and T cell immunity. J Clin Invest.

[66] Bui TM, Wiesolek HL, Sumagin R (2020). ICAM-1: A master regulator of cellular responses in inflammation, injury resolution, and tumorigenesis. J Leukocyte Biol.

[67] Wagner C, Burger A, Radsak M, Blum S, Hug F, Hansch GM (2000). Fibronectin synthesis by activated T lymphocytes: up-regulation of a surface-associated isoform with signalling function. Immunology.

[68] Kudo A, Kii I (2018). Periostin function in communication with extracellular matrices. J Cell Commun Signal.

[69] Huang HH, Bhat A, Woodnutt G, Lappe R (2010). Targeting the ANGPT-TIE2 pathway in malignancy. Nat Rev Cancer.

[70] Sahin U, Oehm P, Derhovanessian E, Jabulowsky RA, Vormehr M, Gold M, Maurus D, Schwarck-Kokarakis D, Kuhn AN, Omokoko T (2020). An RNA vaccine drives immunity in checkpoint-inhibitor-treated melanoma. Nature.

[71] Kubler H, Scheel B, Gnad-Vogt U, Miller K, Schultze-Seemann W, Vom Dorp F, Parmiani G, Hampel C, Wedel S, Trojan L (2015). Self-adjuvanted mRNA vaccination in advanced prostate cancer patients: a first-in-man phase I/IIa study. J Immunother Cancer.

[72] Wildes TJ, Dyson KA, Francis C, Wummer B, Yang C, Yegorov O, Shin D, Grippin A, Dean BD, Abraham R (2020). Immune escape after adoptive T-cell therapy for malignant gliomas. Clin Cancer Res.

[73] Ogando-Rivas E, Castillo P, Jones N, Trivedi V, Drake J, Dechkovskaia A, Candelario KM, Yang C, Mitchell DA (2022). Effects of immune checkpoint blockade on antigen-specific CD8(+) T cells for use in adoptive cellular therapy. Microbiol Immunol.

