# Supplementary material for: mRNA-based precision targeting of neoantigens and tumor-associated antigens in malignant brain tumors
Source: Genome Med. 2024 Jan 25;16:17. doi: 10.1186/s13073-024-01281-z (PMC10809449; doi:10.1186/s13073-024-01281-z)
Supplement: Supplementary file 1 — Additional file 1. This file includes all the supplementary figures and tables described in this manuscript. [file 13073_2024_1281_MOESM1_ESM.docx]

**mRNA-based precision targeting of neoantigens and tumor-associated antigens in malignant brain tumors**

Vrunda Trivedi, Changlin Yang, Kelena Klippel, Oleg Yegorov, Christina von Roemeling, Lan Hoang-Minh, Graeme Fenton, Elizabeth Ogando-Rivas, Paul Castillo, Ginger Moore, Kaytora Long-James, Kyle Dyson, Bently Doonan, Catherine Flores, Duane A. Mitchell

**Corresponding Author-** Duane A. Mitchell, MD, Ph.D., 1333 Center Drive, BSB B1-118, Gainesville, Florida 32610 (email: [Duane.Mitchell@neurosurgery.ufl.edu](mailto:Duane.Mitchell@neurosurgery.ufl.edu); Ph: +1-352-273-9000)

**Additional file 1: Supplementary figures and tables**


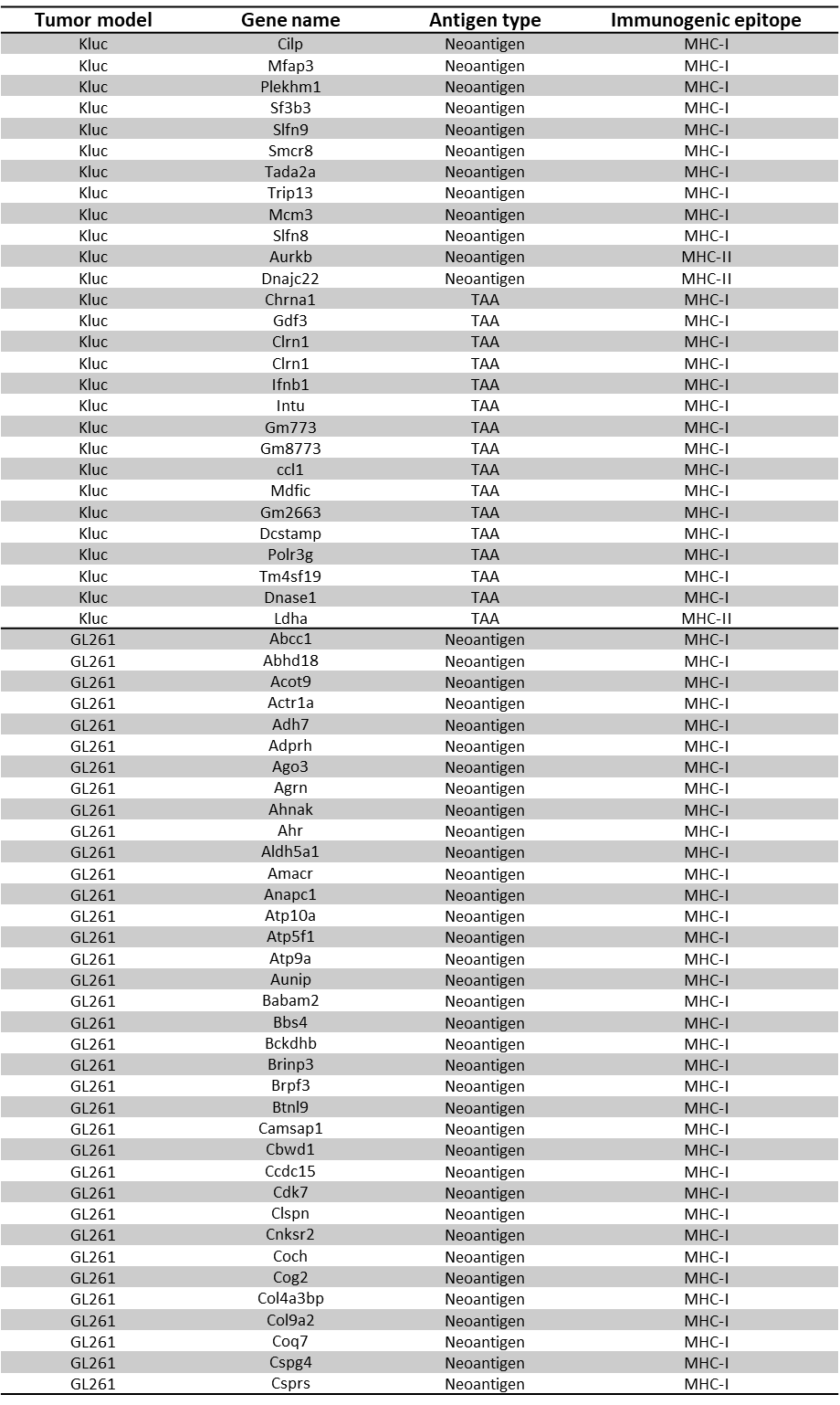


Table S1.


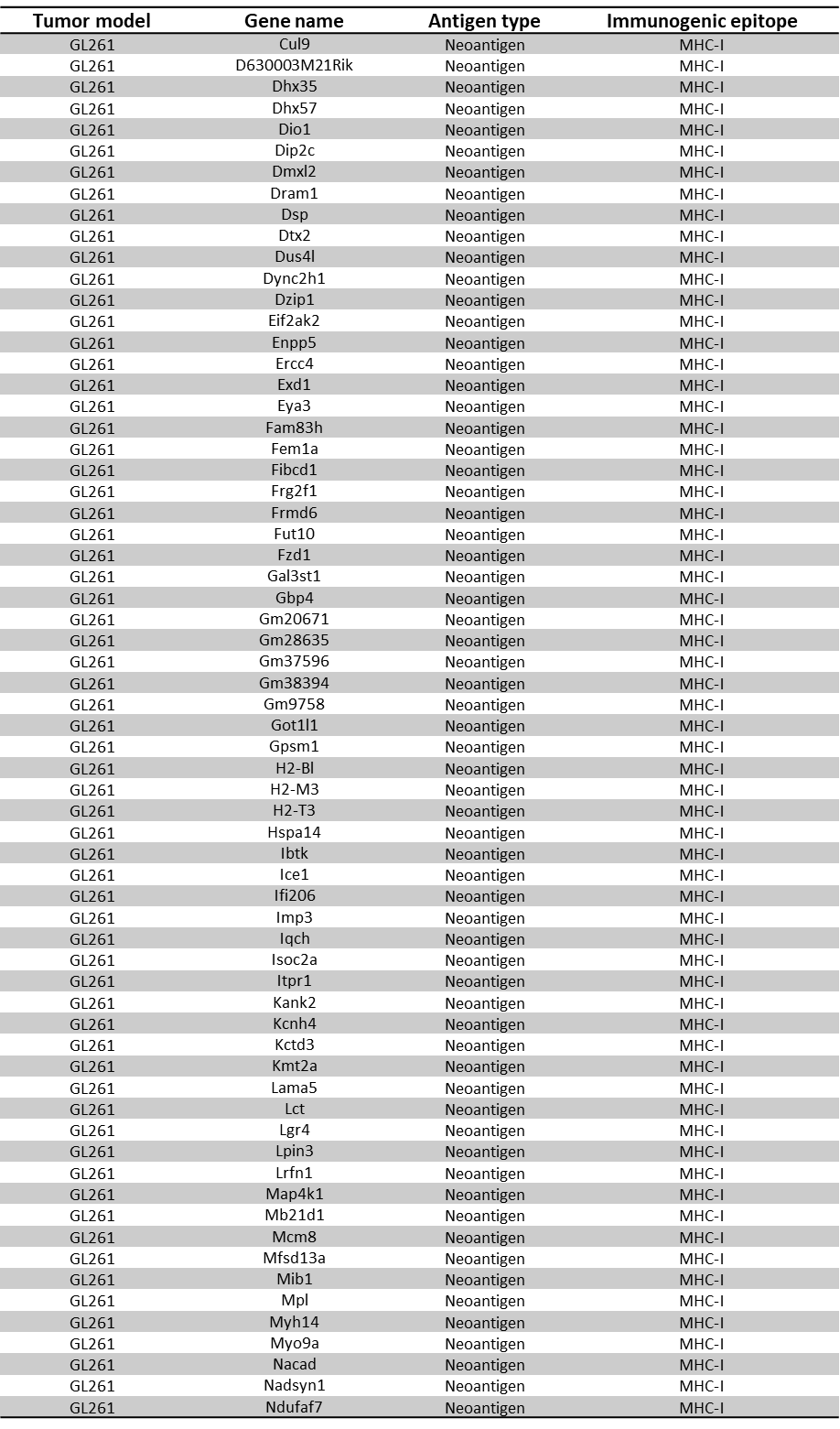


Table S1. continued


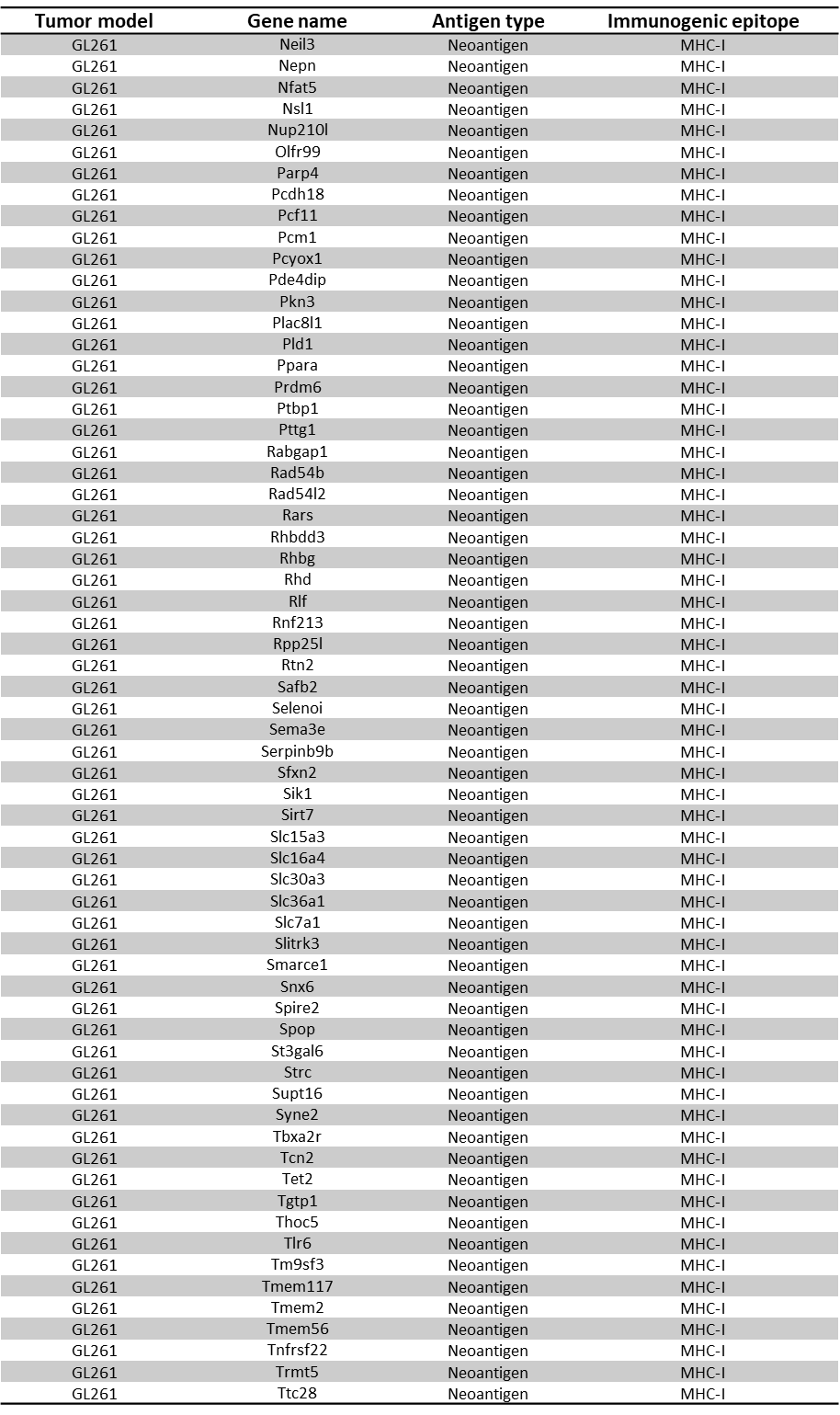


Table S1. continued


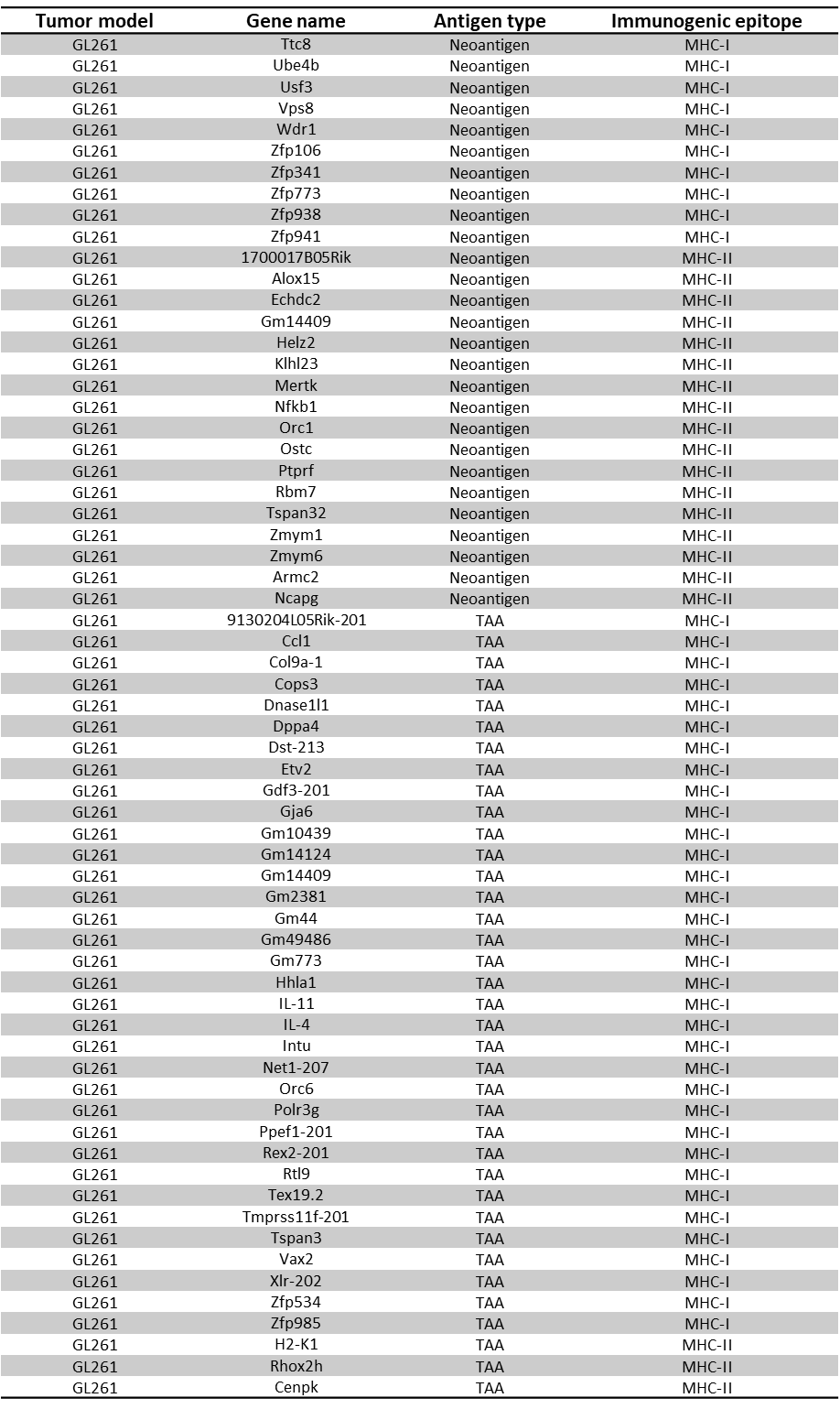


**Table S1: Antigen prediction for murine GBM tumors.** Predicted neoantigens and TAAs for murine GBM tumors based on their affinity for MHC-I or –II molecules using the O.R.A.N. pipeline**.**


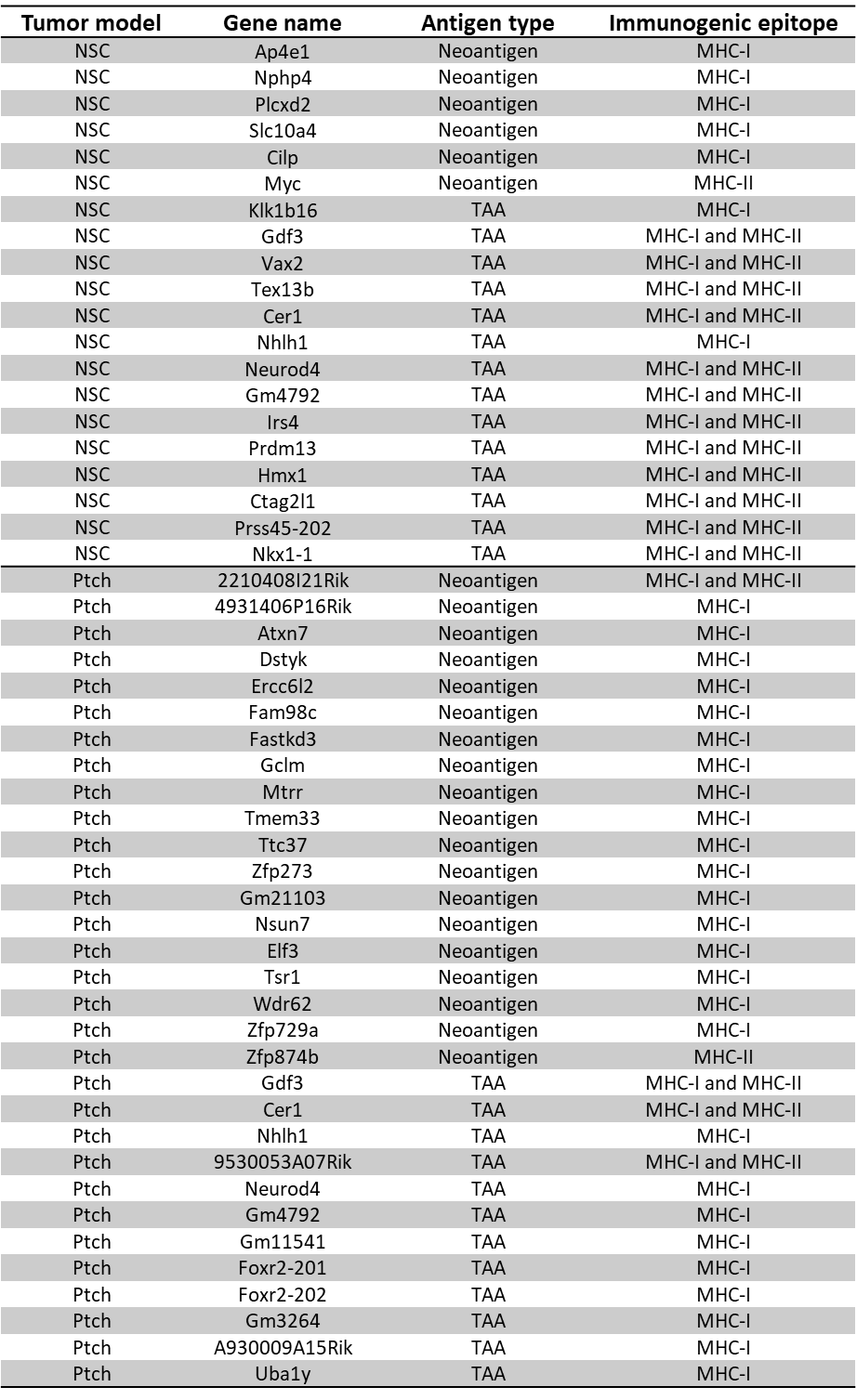


**Table S2: Antigen prediction for murine MB tumors.** Predicted neoantigens and TAAs for murine MB tumors based on their affinity for MHC-I or –II molecules using the O.R.A.N. pipeline**.**

**Figure S1: Antigen prediction using O.R.A.N. algorithm. (a)** A simplified workflow of the O.R.A.N. algorithm that predicts neoantigens and TAAs. **(b)** The average number of total mutations, non-synonymous protein-coding mutations, and immunogenic neoepitopes identified for Kluc (red), GL261 (blue), NSC (green), and Ptch (grey) using the O.R.A.N. pipeline (n=3). **(c)** The average number of tumor-associated genes, and immunogenic TAAs identified for Kluc (red), GL261 (blue), NSC (green), and Ptch (grey) using the O.R.A.N. pipeline (n=3). The antigens are broken down into MHC I- or MHC II- associated epitopes. Some mutations or genes have epitopes that bind both MHC I and II molecules. **(d)** The average mutation burden and tumor-associated genes, and the corresponding neoantigens and TAAs predicted for human GBM tumor samples (n=5 individual patient tumor samples).

**d**

**a**

**b**

**c**


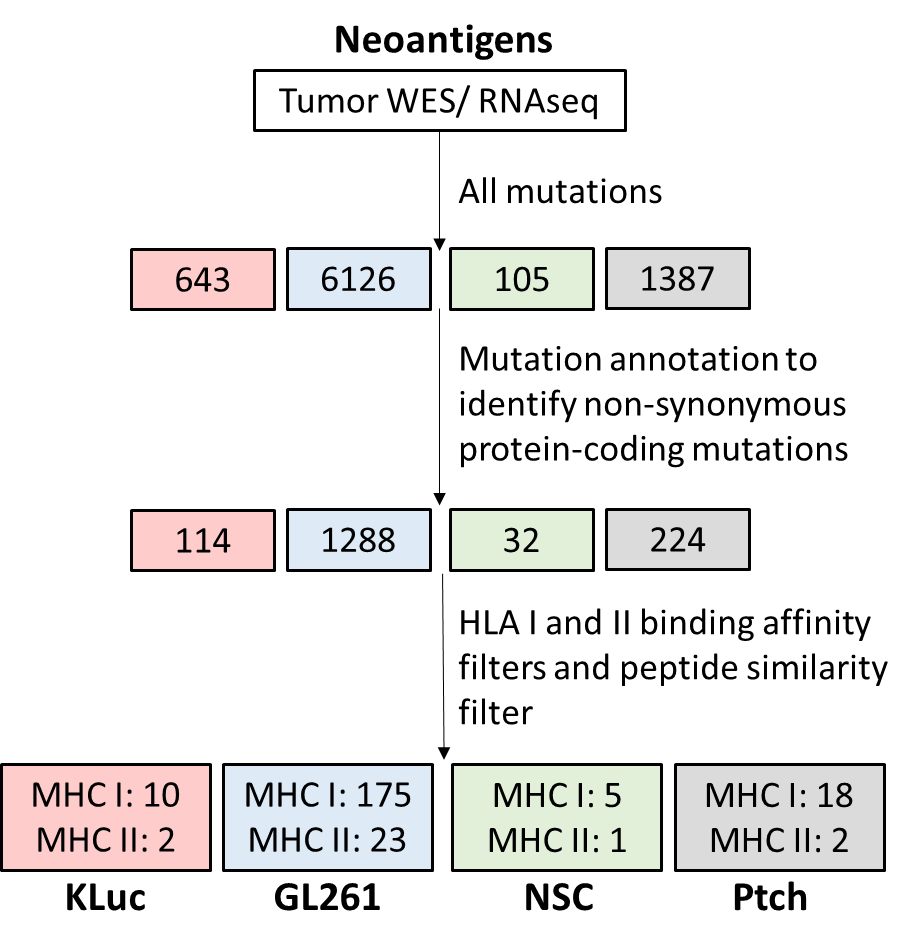

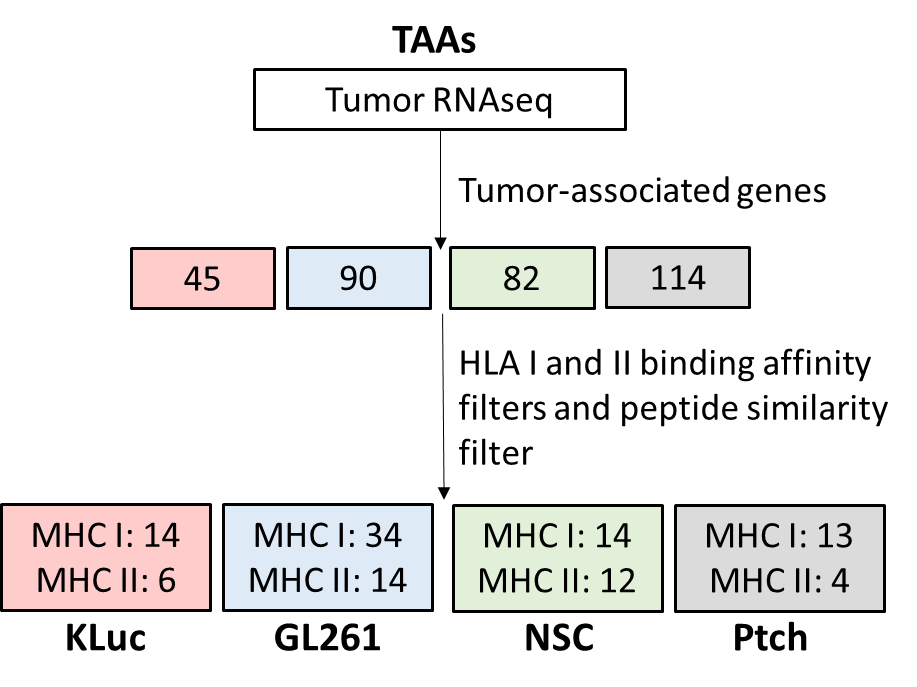

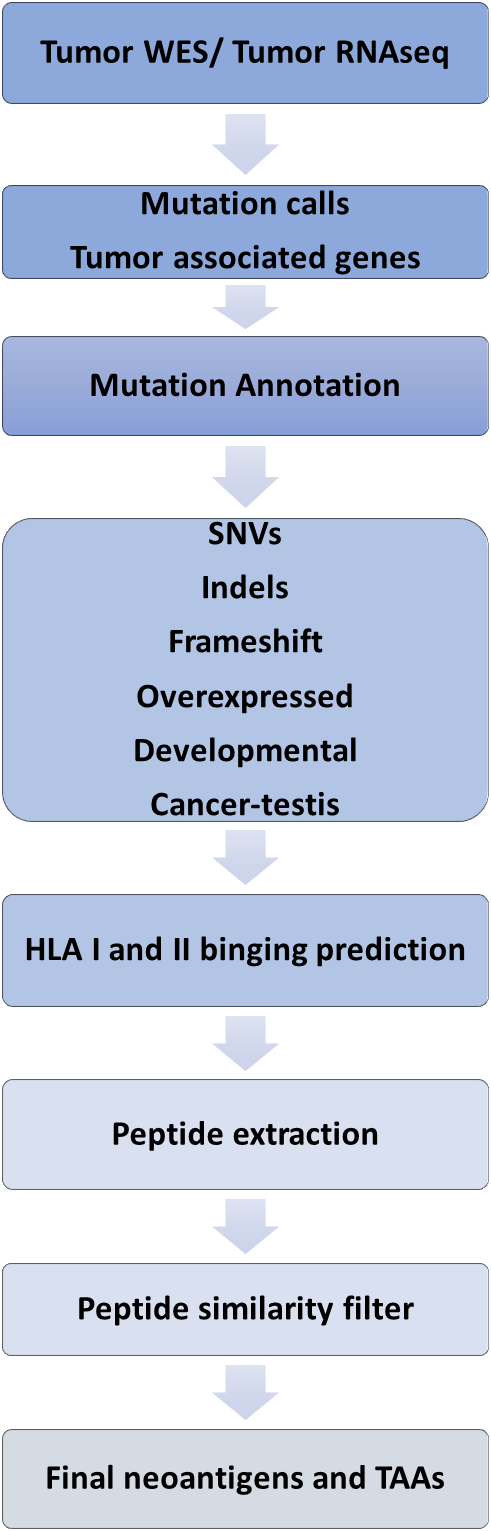


**e**

**f**

**g**


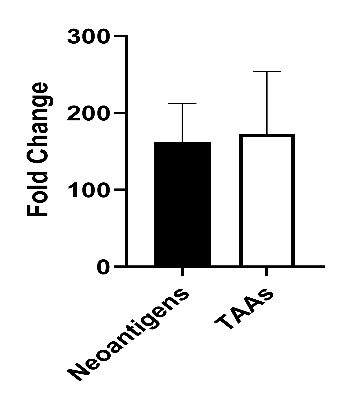


**Kluc**


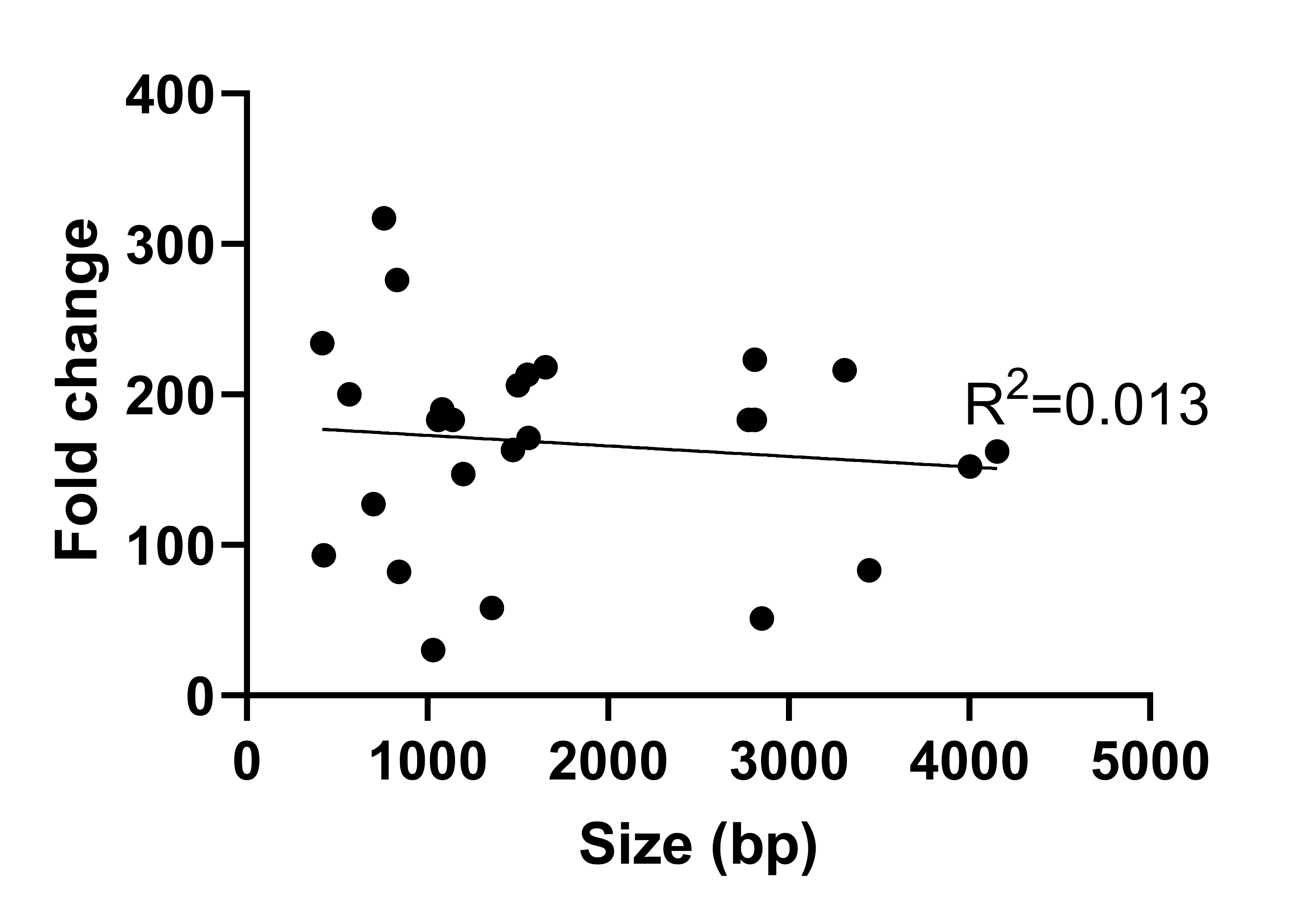


**Kluc**

**Kluc**


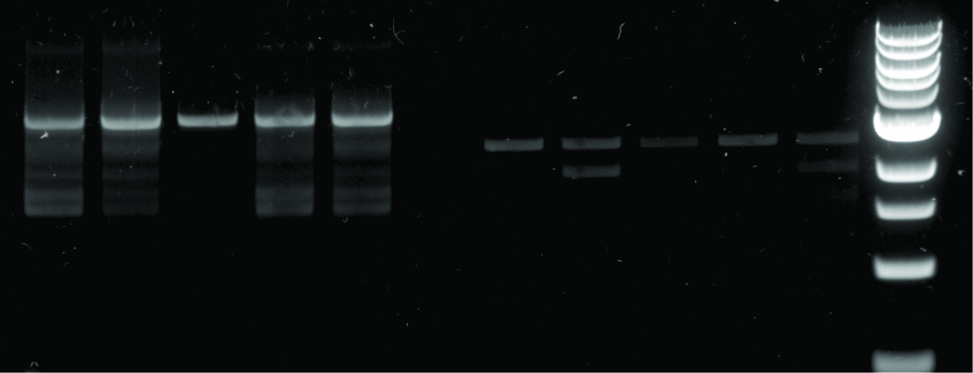


**Cilp (4004 bp)**

**Smcr8 (2808 bp)**

**pre**

**post**

**post**

**pre**

**post**

**post**


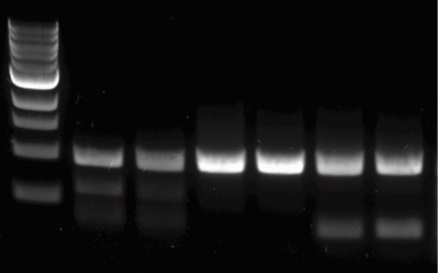


**Neurod4 (971 bp)**

**pre**

**post**

**NSC**


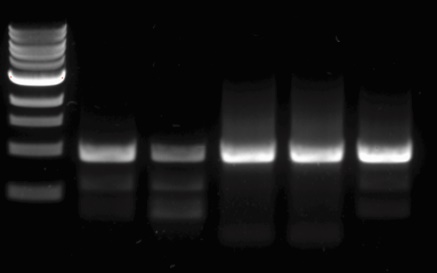


**Neurod4 (971 bp)**

**pre**

**post**

**Ptch**

**h**

**a**

**b**


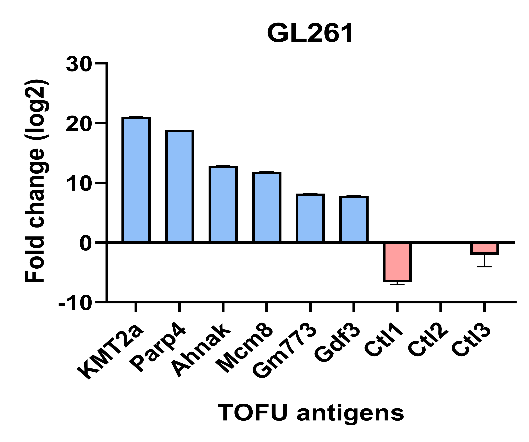

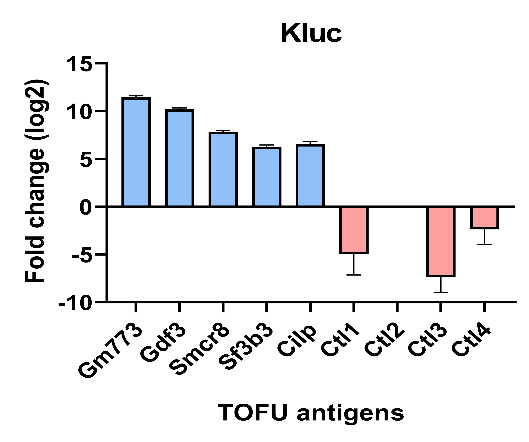

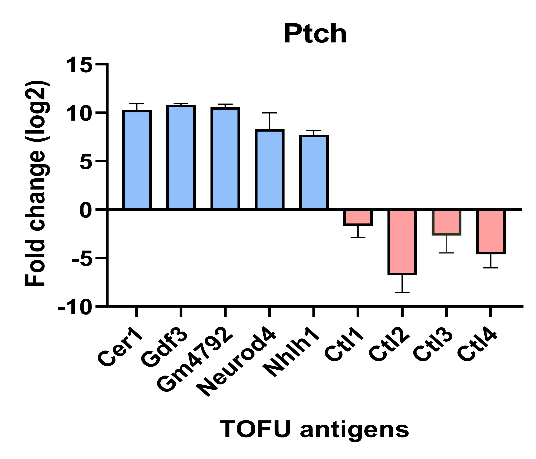

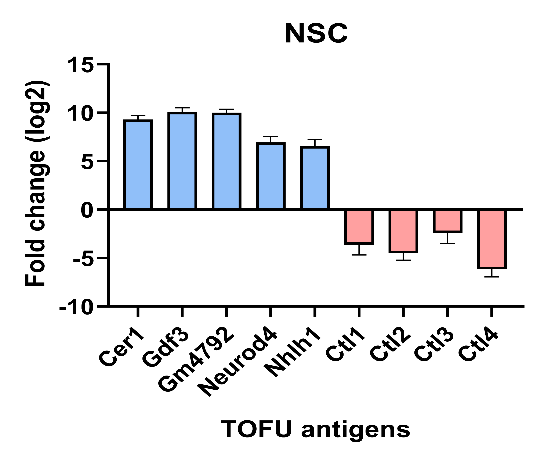


**c**

**d**

**f**

**g**


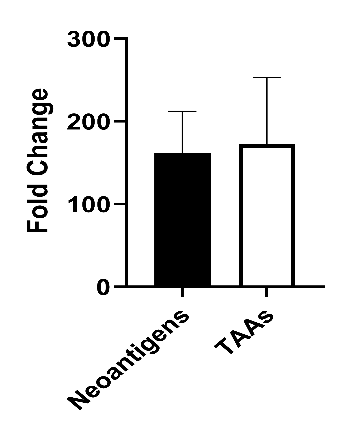


**Kluc**


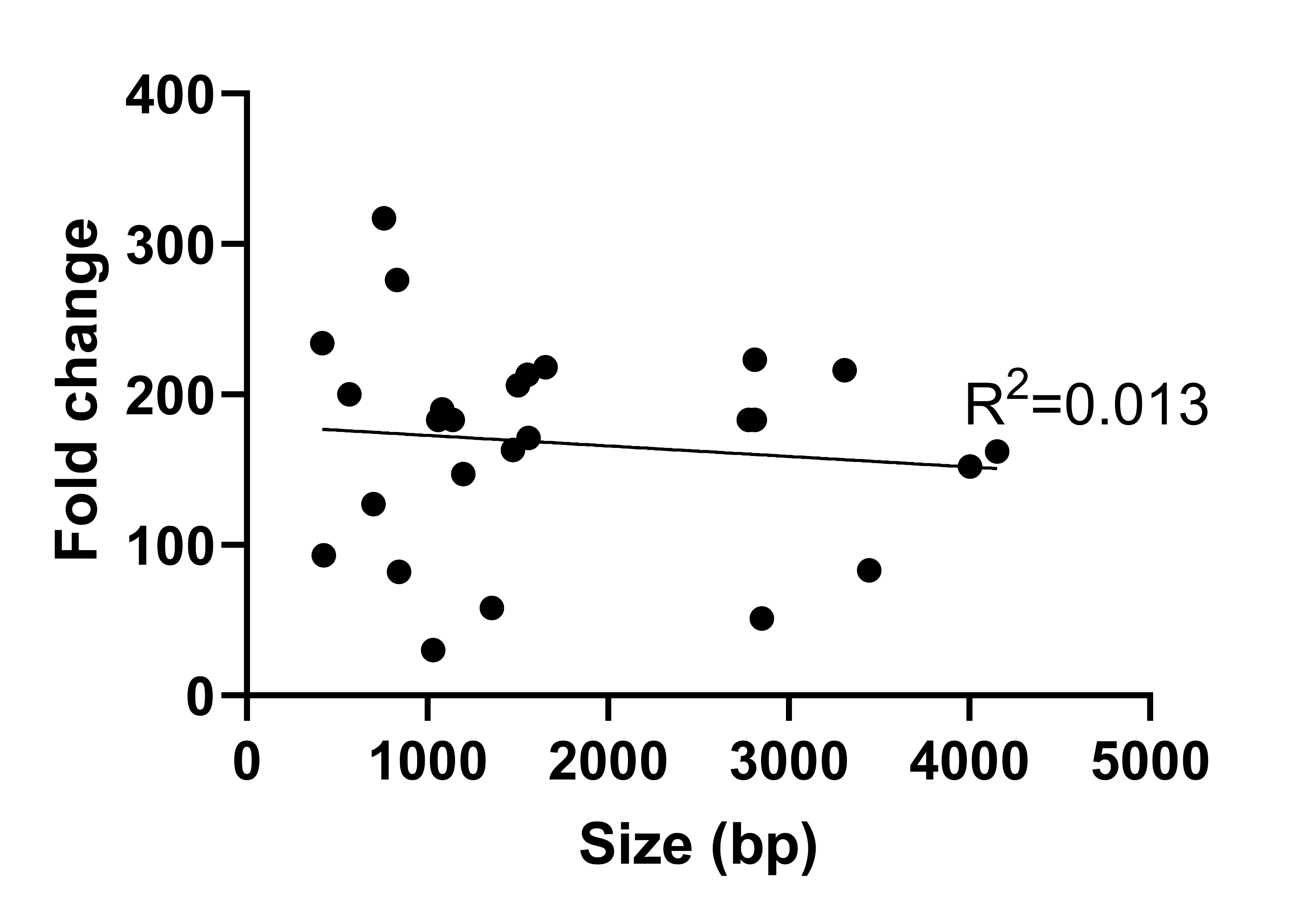


**Kluc**


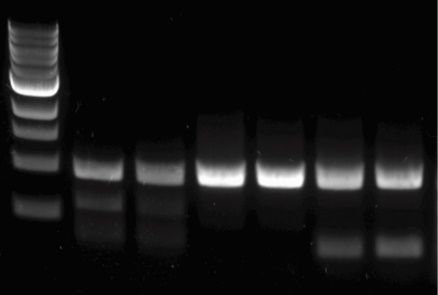


**Neurod4 (971 bp)**

**pre**

**post**

**NSC**


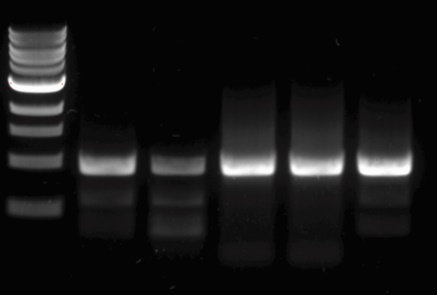


**Neurod4 (971 bp)**

**pre**

**post**

**Ptch**

**h**

**Figure S2: Validation of the antigen enrichment in TOFU mRNA. (a-d)** Fold change increase in the expression of TOFU antigens following the selective gene enrichment strategy as quantified using qPCR. Kluc **(a)**, GL261 **(b)**, NSC **(c)**, and Ptch **(d)** tumor antigens were quantified in the post-selection TOFU mRNA pool as compared to the ttRNA pool. **(e-f)** The capture of full-length mRNA products was verified using PCR analysis of two TOFU antigens in the Kluc tumor model **(e)** and one shared TAA in the NSC and Ptch tumor models **(f)**. **(g)** Comparison of fold change increase in the expression of Kluc neoantigens vs. TAAs. **(h)** Graphical representation of fold change increase in the expression of Kluc TOFU antigens vs. the size of the antigen sequences.


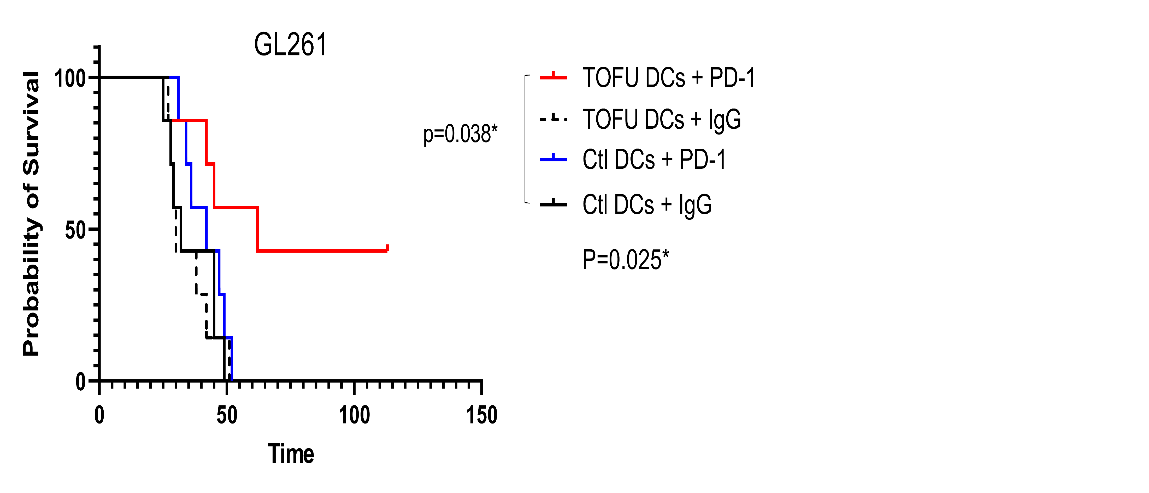

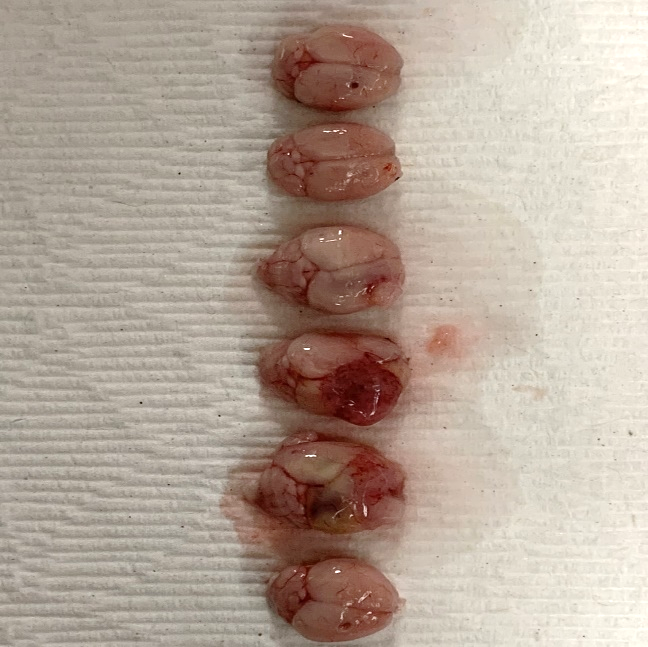

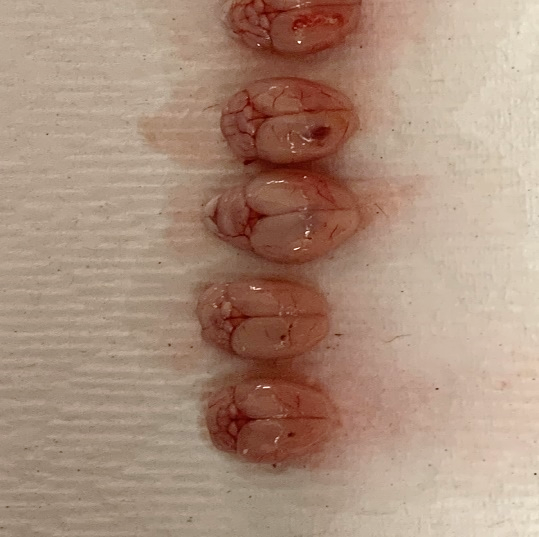

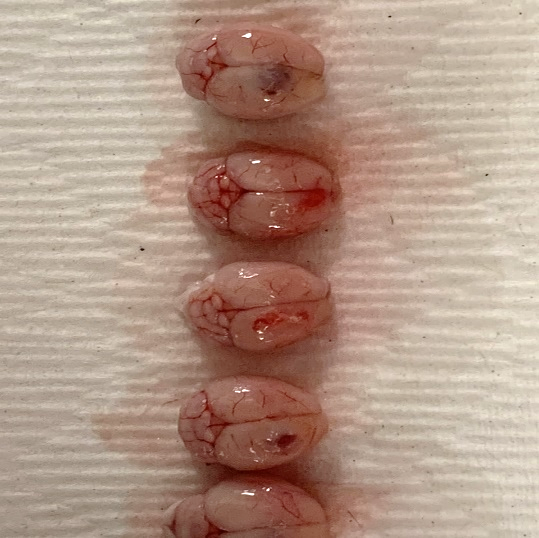

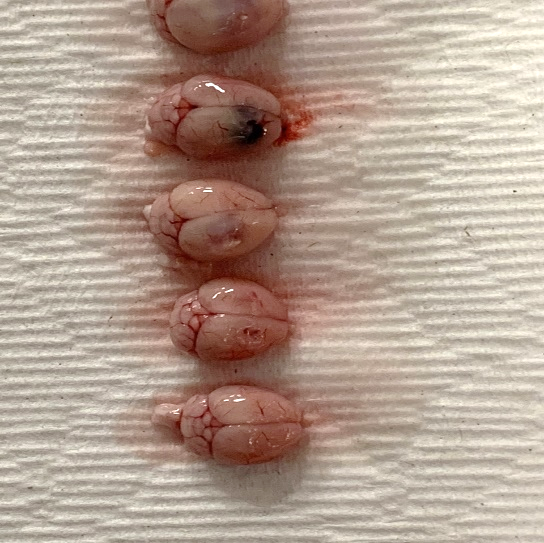

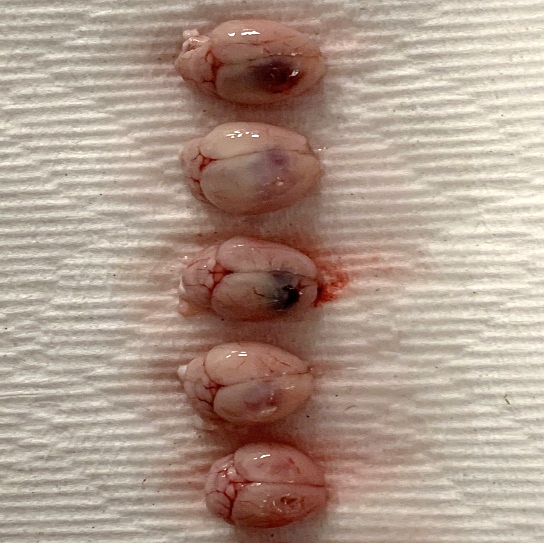

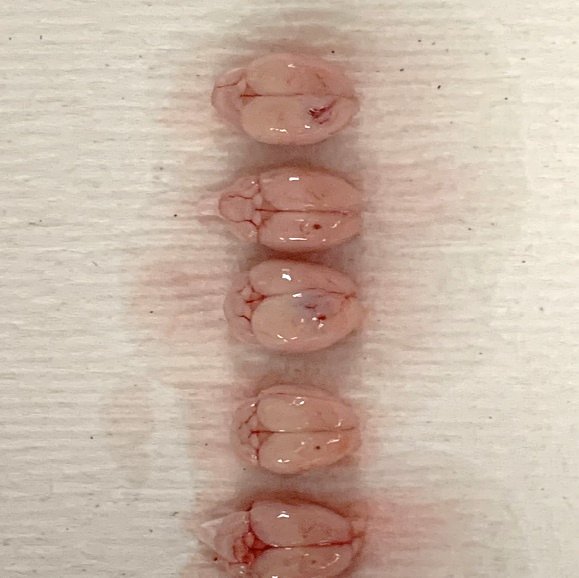

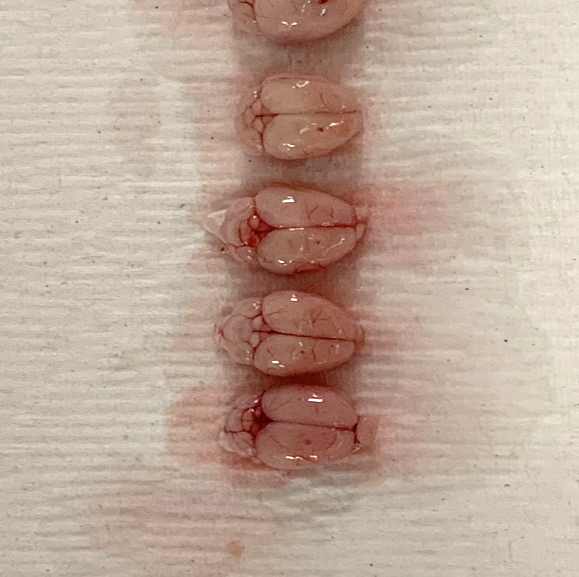


**Ctl DCs+ IgG**

**Ctl DCs+ PD-1**

**Gl261 DCs + IgG**

**GL261 DCs+ PD-1**

**a**

**b**


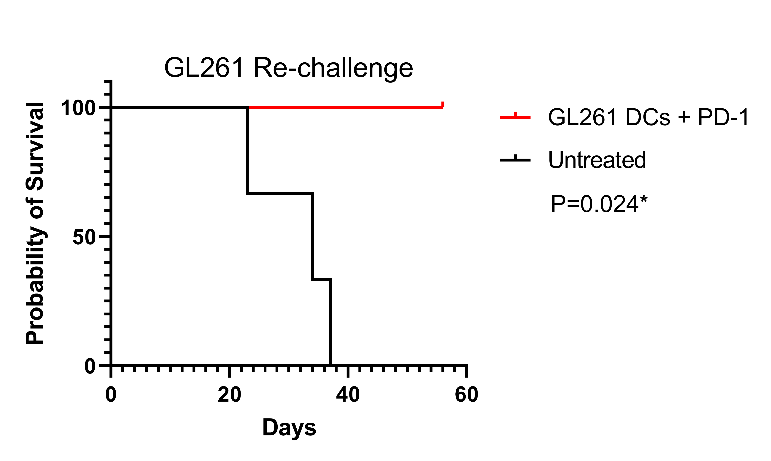


**Figure S3: Therapeutic efficacy of TOFU mRNA vaccine in combination with ICIs. (a)** ImageJ software analysis of the tumor area measurement in brain tissue isolated following TOFU DCs+PD-1 and control treatments in GL261 tumor-bearing mice. **(b)** Survival curve of the TOFU DCs+PD-1 treated mice upon re-challenge with GL261 tumors as compared to untreated mice (n=3 per group). Statistical analysis was performed using the log-rank (Mantel-Cox) test with significance at p<0.05.


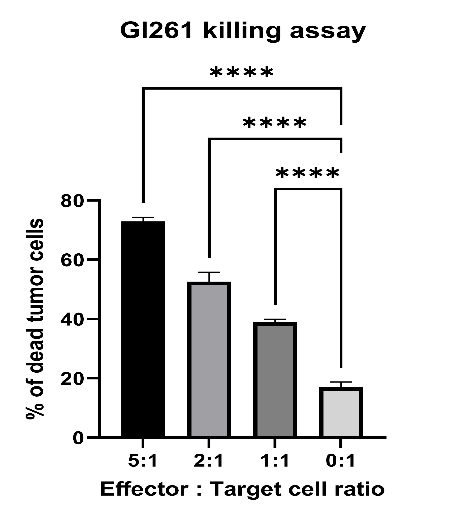

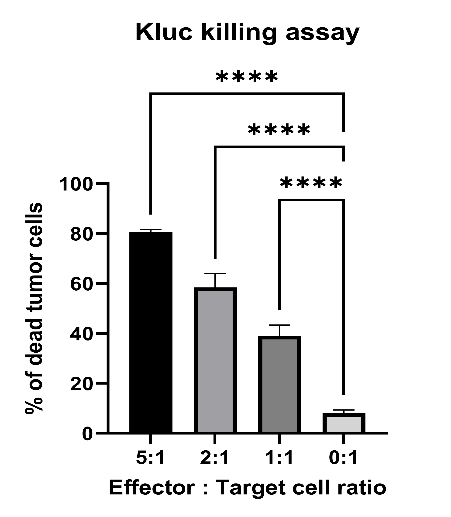

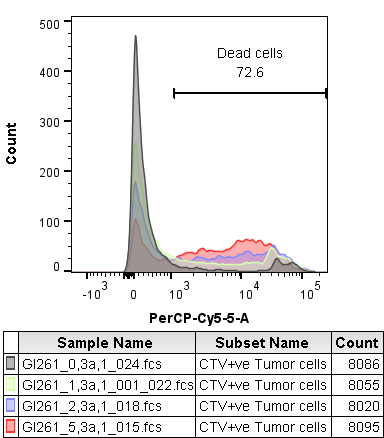

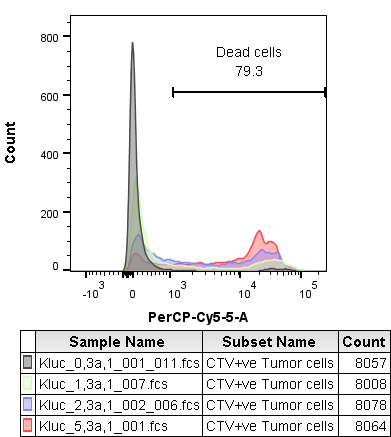


**c**

**b**

**a**


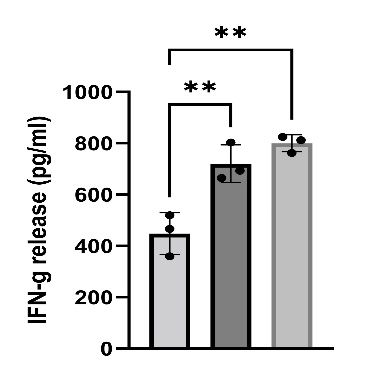


**Kluc TOFU T cells**

+ + +

**DCs + GFP RNA**

+ - -

**DCs + TOFU RNA**

- + -

**Kluc tumor cells**

- - +

**Figure S4: Validation of TOFU mRNA vaccine specificity to the tumor. (a)** Reactivity of the TOFU mRNA-specific *ex vivo* expanded T-cells to Kluc tumor cells and target cells loaded with TOFU-mRNA or GFP RNA. IFNγ secretion as measured by ELISA after 48 h of co-culture (n=3 per group). Statistical analysis was done using one-way ANOVA and Tukey’s multiple comparisons. **(b-c)** Flow cytometry analysis of live/dead tumor cells following co-culture with *ex vivo* expanded TOFU mRNA-specific T-cells in different effector: target cell ratios in Kluc **(b)** and GL261 **(c)** models. Statistical analysis was done using one-way ANOVA and Tukey’s multiple comparisons. p<0.05 is *, p<0.01 is **, p <0.001 is ***, and p<0.0001 is ****.


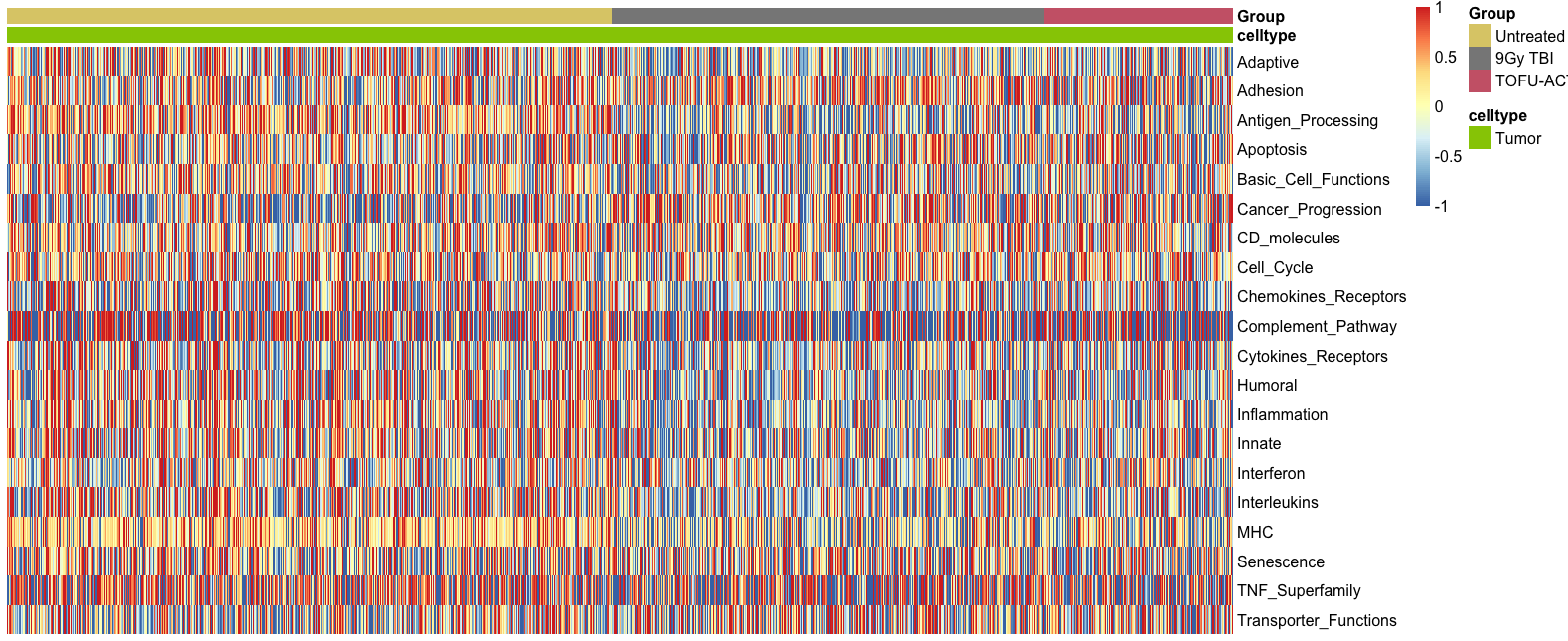


**a**


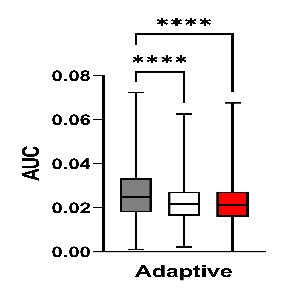

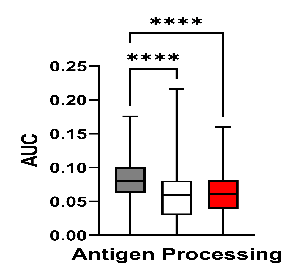

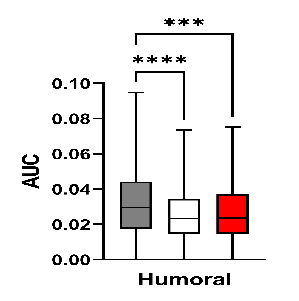

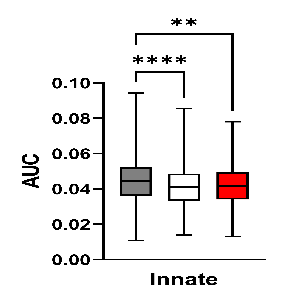

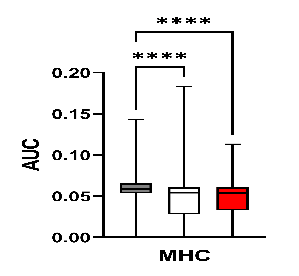

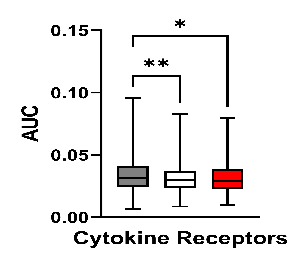

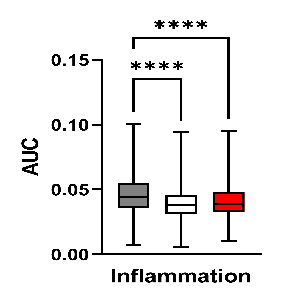

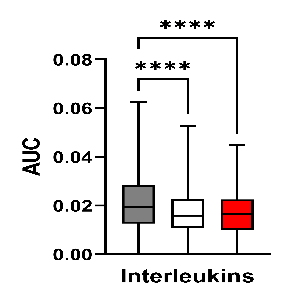

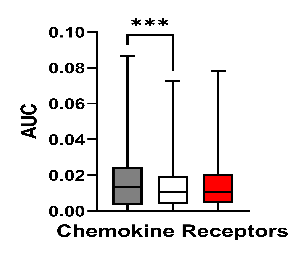

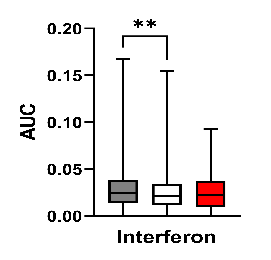

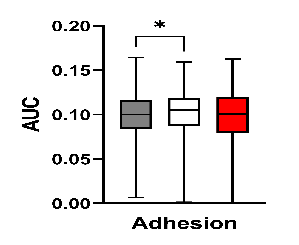

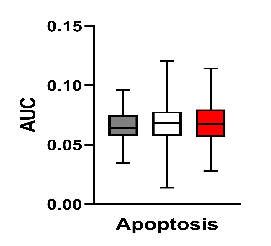

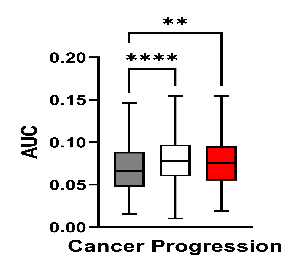

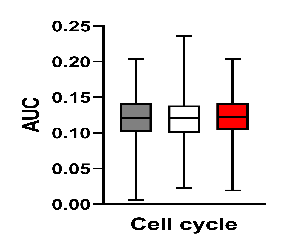

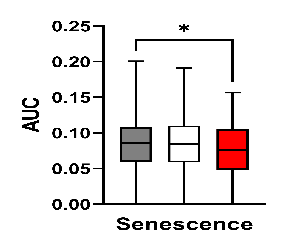


**b**

**Figure S5: Tumor cell gene signature alteration following the TOFU-ACT treatment. (a)** Heatmap representation of pathway-based gene expression analysis of the luciferase +ve Kluc tumor cells using the nCounter PanCancer panel from NanoString following treatment with TOFU-ACT. **(b)** Quantification of the pathway analysis in (a). Statistical analysis was performed using Kruskal-Wallis and Dunn’s test for multiple comparisons; p<0.05 is *, p<0.01 is **, p <0.001 is ***, and p<0.0001 is ****.


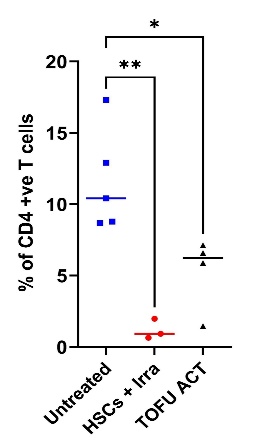

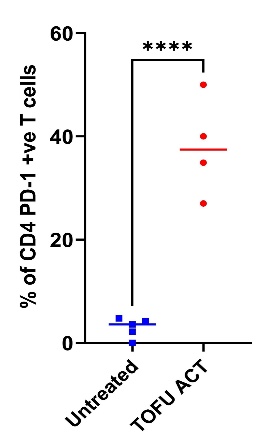

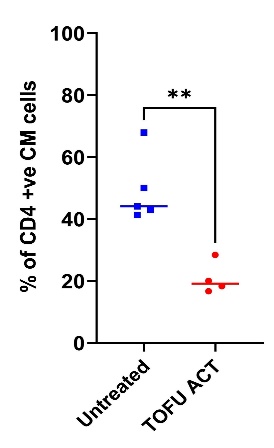

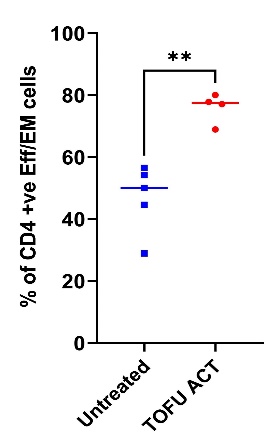

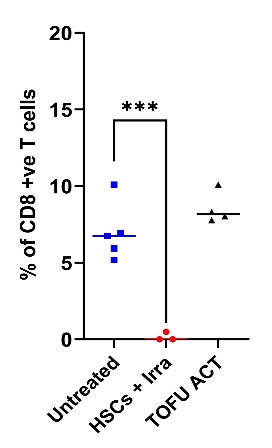

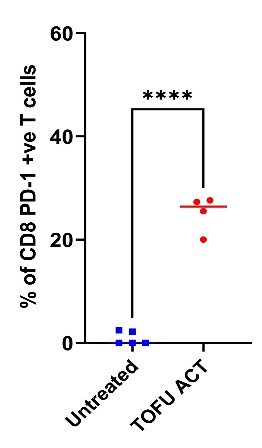

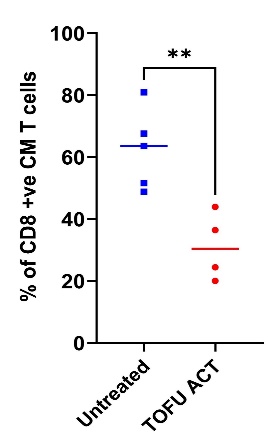

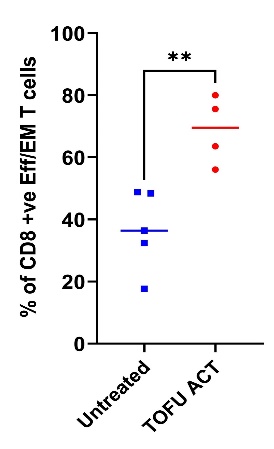


**a**

**b**

**Figure S6: Peripheral T-cell phenotyping following the TOFU-ACT treatment. (a-b)** Flow cytometry analysis of peripheral CD4+ **(a)** and CD8+ **(b)** T-cell phenotype in the TOFU-ACT treated or untreated Kluc tumor-bearing mice (n=4 to 5 per group). Statistical analysis was done using one-way ANOVA with Tukey’s multiple comparisons, or individual student t-tests; p<0.05 is *, p<0.01 is **, p <0.001 is ***, and p<0.0001 is ****.


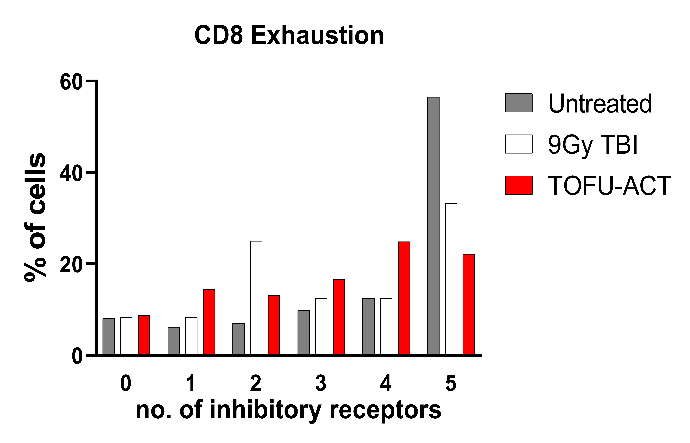

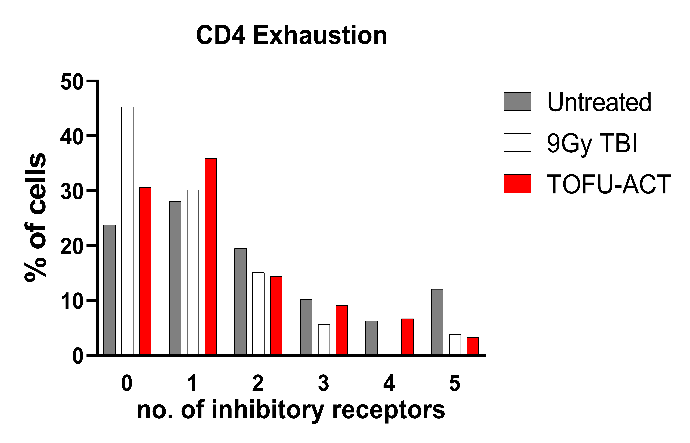

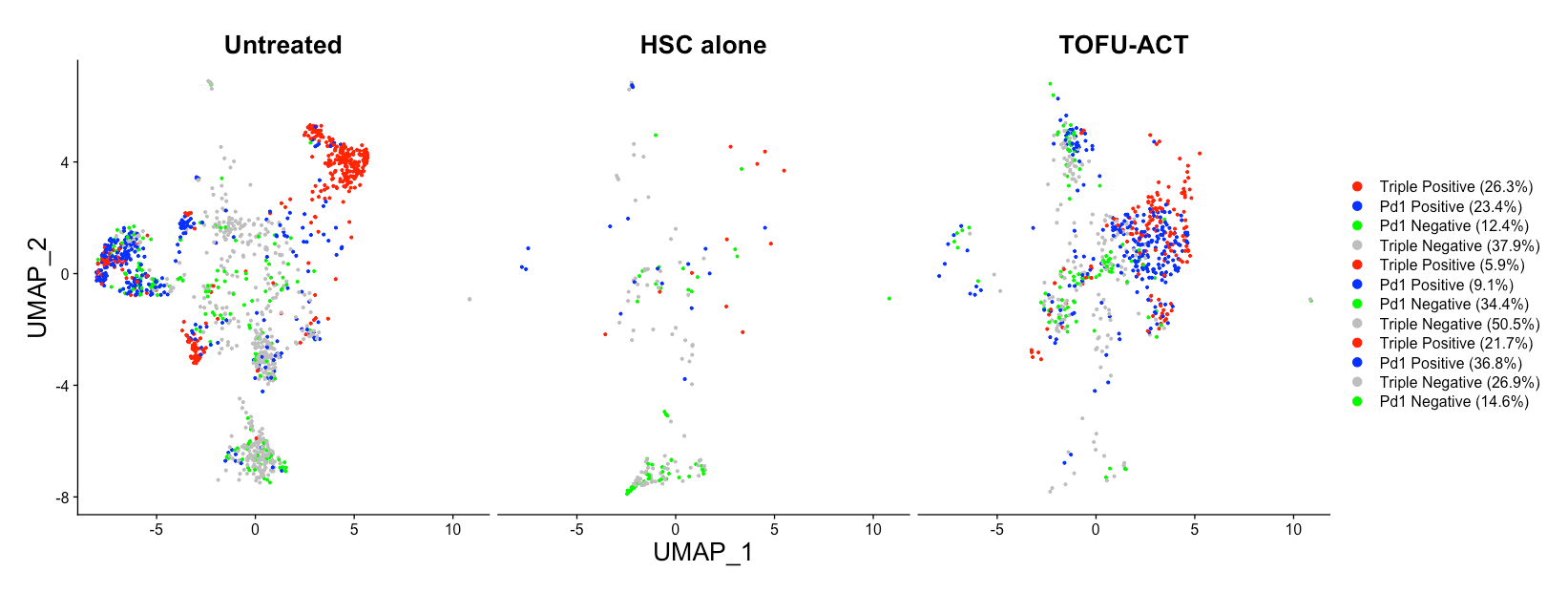

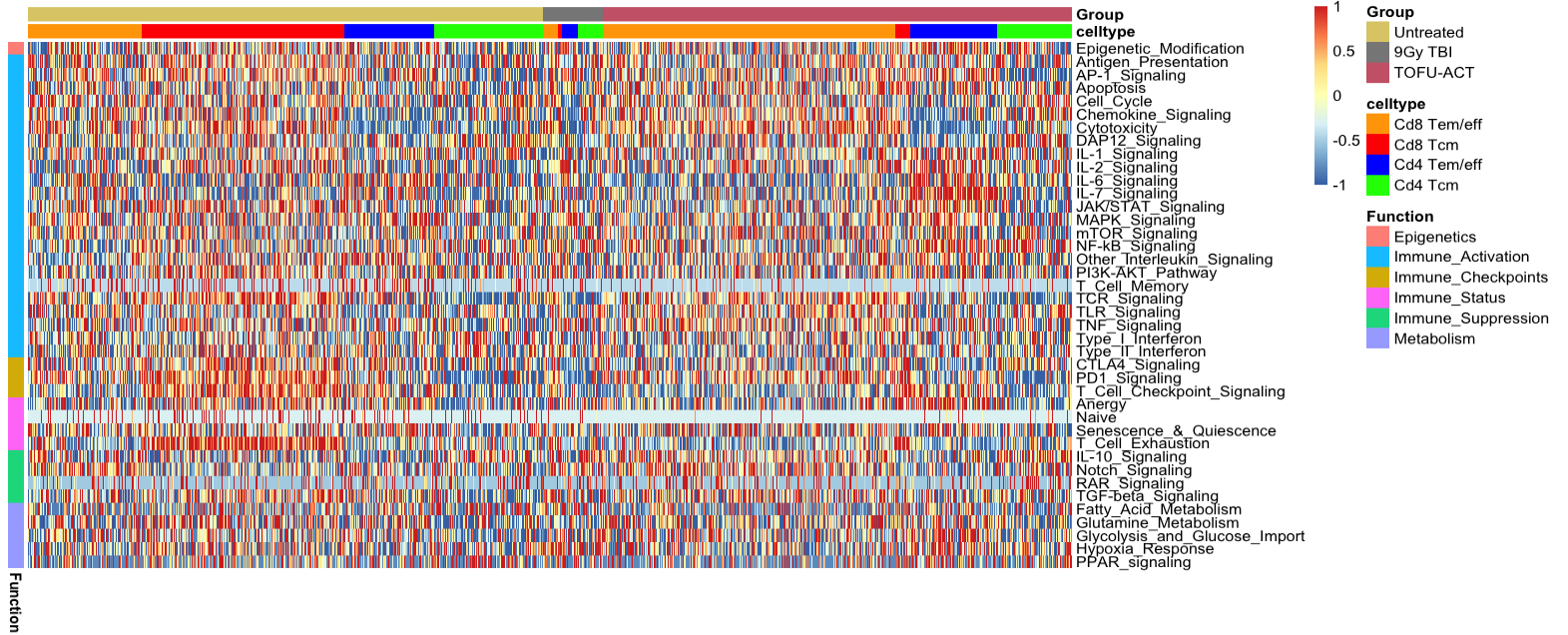


**a**

**b**

**c**


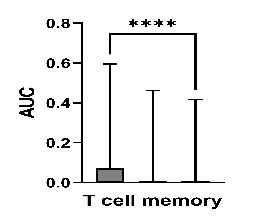

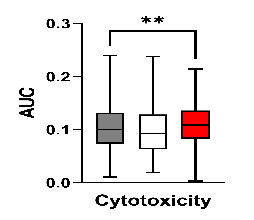

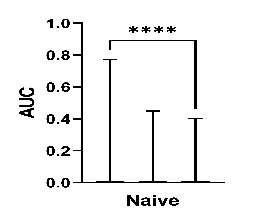

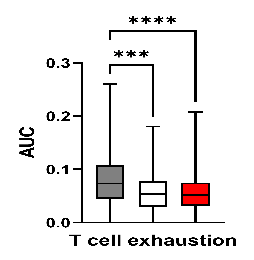

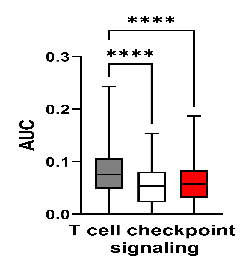


**d**

**e**

**Figure S7: T-cell exhaustion and gene expression analysis following the TOFU-ACT treatment. (a-b)** Percentage of cells co-expressing exhaustion markers Pdcd1, Tigit, Lag3, Havcr2, and CTLA4, with 0 being no expression and 5 being all 5 receptors expressed. **(a)** shows CD8+ T-cells and **(b)** shows CD4+ T-cells. **(c)** UMAP showing the projection of triple positive (Pdcd1+, Lag3+, and Havcr2), Pdcd+, Pdcd-, or triple-negative T-cells. **(d)** Heatmap representation of pathway-based gene expression analysis of the T-cells using the nCounter Immune Exhaustion Pathways panel from NanoString following treatment with TOFU-ACT. **(e)** Quantification of the gene expression in (d) for pathways of interest. Statistical analysis was performed using Kruskal-Wallis and Dunn’s test for multiple comparisons; p<0.05 is *, p<0.01 is **, p <0.001 is ***, and p<0.0001 is ****.


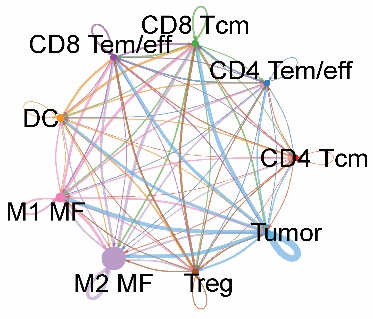


**Untreated**


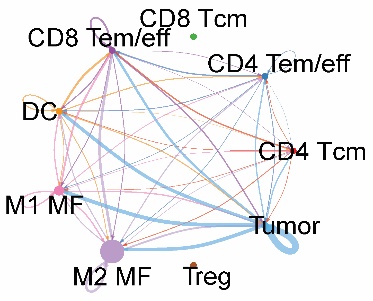


**9Gy TBI**


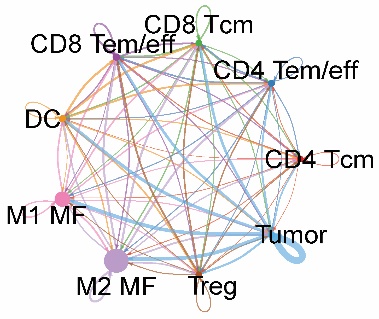


**TOFU-ACT**


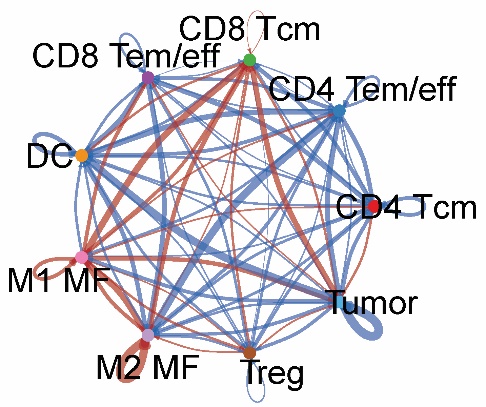

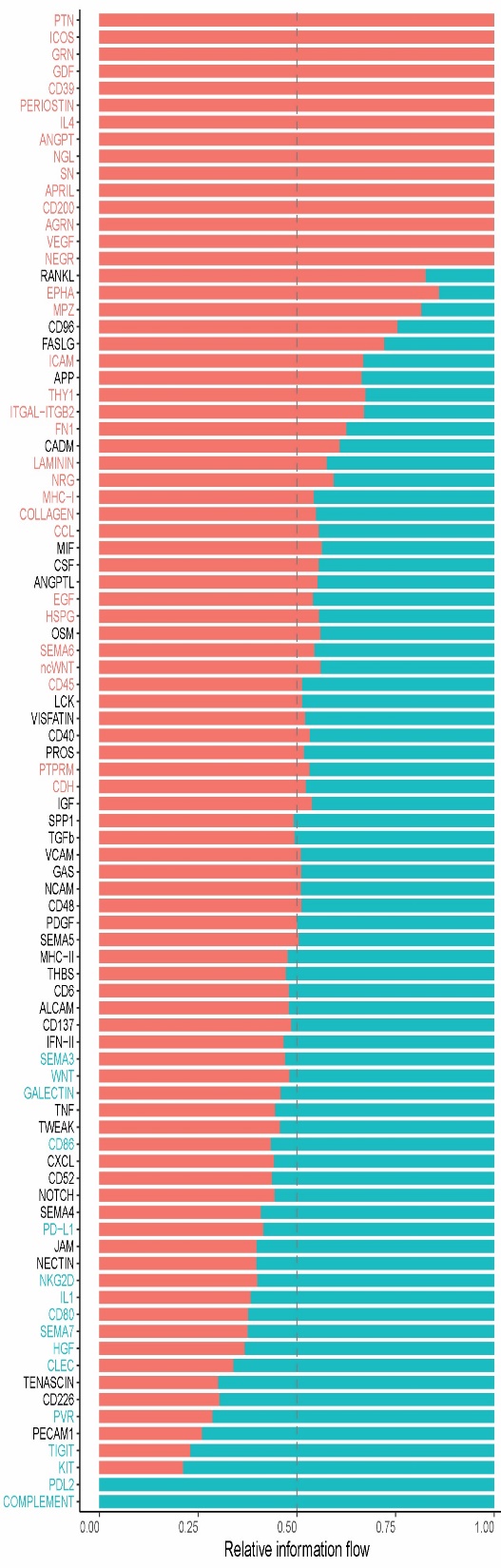

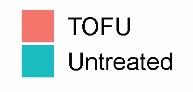


**a**

**b**

**c**

**d**


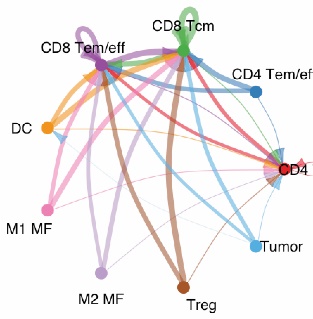

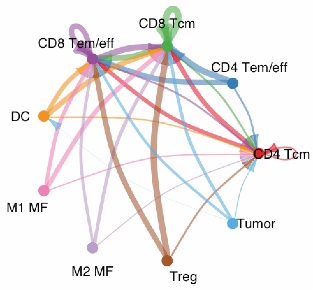

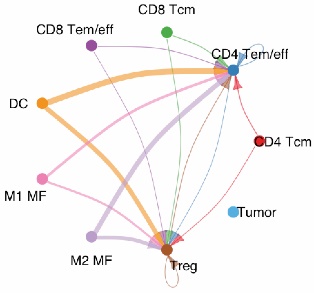

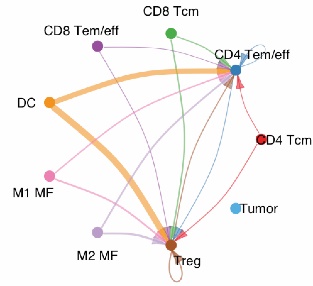


Untreated

TOFU-ACT

**MHC-I interactions**

**MHC-II interactions**

Untreated

TOFU-ACT

**Figure S8: Immune cell-cell interactions within the tumor microenvironment of TOFU-ACT treated mice. (a)** Immune cell interactions are shown using the CellChat algorithm. The connecting lines show the flow of information from senders (ligands) to receivers (receptors) and the thickness of the lines depicts the strength of interactions between any two immune cell populations. **(b)** Changes in the number or strength of immune cell interactions in the TOFU-ACT treated tumors as compared to the untreated ones. The blue color shows the downregulation of interactions between two cell populations while the red color shows the upregulation of interactions. **(c)** Chord plots showing MHC-II and MHC-I specific interactions between the immune cells. **(d)** Relative information flow plot showing the strength of specific interactions in the TOFU-ACT group vs. the untreated group. The data labels in red or blue are significantly different. The ones which are of particular interest are highlighted with black arrows.
